# The Comprehensive Profiling of the Chemical Components in the Raw and Processed Roots of *Scrophularia ningpoensis* by Combining UPLC-Q-TOF-MS Coupled with MS/MS-Based Molecular Networking

**DOI:** 10.3390/molecules29204866

**Published:** 2024-10-14

**Authors:** Mina Zhang, Kaixian Chen, Chenguo Feng, Fang Zhang, Liuqiang Zhang, Yiming Li

**Affiliations:** 1 School of Pharmacy, Shanghai University of Traditional Chinese Medicine, Shanghai 201203, Chinaymli@shutcm.edu.cn (Y.L.); 2 The Research Centre of Chiral Drugs, Innovation Research Institute of Traditional Chinese Medicine, Shanghai University of Traditional Chinese Medicine, Shanghai 201203, China

**Keywords:** *Scrophularia ningpoensis*, TCM processing method, UPLC-Q-TOF-MS, GNPS, processing mechanism

## Abstract

Scrophulariae Radix (SR), the dried root of *Scrophularia ningpoensis* Hemsl (*S. ningpoensis*), has been extensively used as traditional Chinese medicine for thousands of years. However, since the mid-20th century, the traditional processing technology of *S. ningpoensis* has been interrupted. Therefore, ultra-high performance liquid chromatography coupled with quadrupole time-of-flight mass spectrometry technology, together with a Global Natural Product Social Molecular Networking (GNPS) method, was applied to comprehensively analyze the characteristic changes and mutual transformation of chemical constituents in the differently processed roots of *S. ningpoensis*, as well as to scientifically elucidate the processing mechanism of differently processed SR. As a result, a total of 149 components were identified. Notably, with the help of the GNPS data platform and MS^2^ fragment ions, the possible structures of four new compounds (**47**, **48**, **50**, and **73**) were deduced in differently processed SR samples, in which **47**, **48**, and **50** are iridoid glycosides, and **73** is a phenylpropanoid glycoside. Five cyclopeptides (**78**, **86**, **97**, **99**, and **104**) derived from leucine (isoleucine) were identified in SR for the first time. The heatmaps analysis results indicated that leucine or isoleucine may be converted to cyclopeptides under the prolonged high-temperature conditions. Moreover, it is found that short-time steaming can effectively prevent the degradation of glycosides by inactivating enzymes. This study provides a new and efficient technical strategy for systematically identifying the chemical components, rapidly discovering the components, and preliminarily clarifying the processing mechanism of *S. ningpoensis*, as well as also providing a scientific basis for the improvement of the quality standards and field processing of *S. ningpoensis.*

## 1. Introduction

Scrophulariae Radix (SR), the dried root of *Scrophularia ningpoensis* Hemsl, has been extensively used in traditional Chinese medicine (TCM). SR was first reported in Shennong’s classic work of Materia Medica. According to the conventional TCM theories, SR provides various benefits, such as reducing heat to cool blood, nourishing yin, reducing pathogenic heat, detoxification, and resolving hard masses. Modern pharmacology has identified its anti–inflammatory [1,2], neuroprotective [1,3], cardioprotective [4], antiallergenic [5], antiprotozoal, and antimicrobial effects [6,7]. The primary processing of original materials is crucial to ensure high–quality SR. The “steaming” processing method (TPM) of SR was first recorded in Lei Gong’s Treatise on Preparation and Boiling of Materia Medica during the reign of the Northern and Southern dynasties (AD 420–−589). Until the reign of the Ming and Qing dynasties (approx. AD 1368–−1911), SR was mainly processed by a slicing and baking processing method (BPM) or TPM. According to the Chinese Pharmacopoeia from 1936 to 2020, SR was supposed to be basked or baked until semidry after removing the shoots, fibrous roots, and sediment from the fresh roots; then, the SR should be stacked for 3–6 days, and these steps should be repeated until the SR is completely dry. This method is referred to as the sweating processing method (WPM). However, previous reports have suggested that the WPM of SR has gradually developed over a period of 60 years alongside the long–term practice of primary processing of medicinal materials in producing regions, rather than through a conventional processing method for SR [8]. Meanwhile, it has not been reported how the ancient and modern processing technology affects the intrinsic chemical makers of the raw and processed roots of *S. ningpoensis*.

Recently, with the widespread use of high/ultra–high performance liquid chromatography (HPLC/UHPLC) coupled with various types of mass spectrometers (HPLC/UHPLC–MS^n^), thousands of complex components from natural products (NPs) can be specifically detected to distinguish the raw and processed products of the TCM, or even evaluate the extent of the processing [9,10]. Aside from the measurement of accurate mass and the generation of the MS^n^ data for the better elucidation of the chemical structures, HPLC/UHPLC–MS^n^ has also shown distinct superiority on high–throughput screening and rapid identification using a single injection with less consumption of organic solvents [11,12,13]. Nevertheless, unequivocal identification by HPLC/UHPLC–MS^n^ can be achieved only when the reference compounds are available. Due to the minor trace compounds, isomers, the complexity of targeted components, and diverse structural skeletons, it is complicated and time–consuming to characterize the constituents in NPs [14]. 

Molecular networking (MN), which was introduced in 2012, enabled the analyzing of tandem mass spectra of small molecules and the mapping of the chemical diversity observed in an untargeted mass spectrometry [15,16]. Global Natural Products Social Molecular Networking (GNPS, http://gnps.ucsd.edu (accessed on 2 December 2023)), an open–access knowledge base, provides the ability to deposit, analyze, and disseminate the knowledge of MS/MS spectra that enables the community sharing of raw spectra, the continuous annotation of spectra data, and the collaborative curation of reference spectra and experimental data [17]. Through aligning each MS/MS spectrum in a dataset to each of the others and assigning a cosine score to each combination to describe their similarity, ranging from 0 (totally dissimilar) to 1 (completely identical), the identical masses are collapsed on the basis of a hierarchical cosine clustering algorithm into a single node or consensus cluster via edges because of the high similarity of their fragment ions GNPS [15,18,19]. As a consequence, a visual representation of the structural relationships between natural compounds within crude extracts of TCM is generated, where a node within the network represents a spectrum of the compound and spectrum–to–spectrum alignments as edges (connections) between two nodes [17,20].

In order to systematically investigate the chemical components and explore the processing change rules of various compounds between the raw and processed roots of *S. ningpoensis*, UPLC coupled with quadrupole time–of–flight mass spectrometry (UPLC–Q–TOF–MS) technology and a GNPS data platform were applied to rapidly classify and analyze the various compounds in the raw and processed roots of *S. ningpoensis* based on the concept that compounds with similar structures cluster together in MN spectra. Additionally, the changes in the relative content of the identified compounds in the raw and processed roots of *S. ningpoensis* are discussed by comparing their relative ion abundance, which lays a foundation for further research on the pharmacological activity, –spectrum–effect relationship and quality control, and provides a useful strategy for further optimizing the industrial processing methods of the roots of *S. ningpoensis*.

## 2. Results and Discussion

### 2.1. Identification of the Components from S. ningpoensis

**Figure 1:**
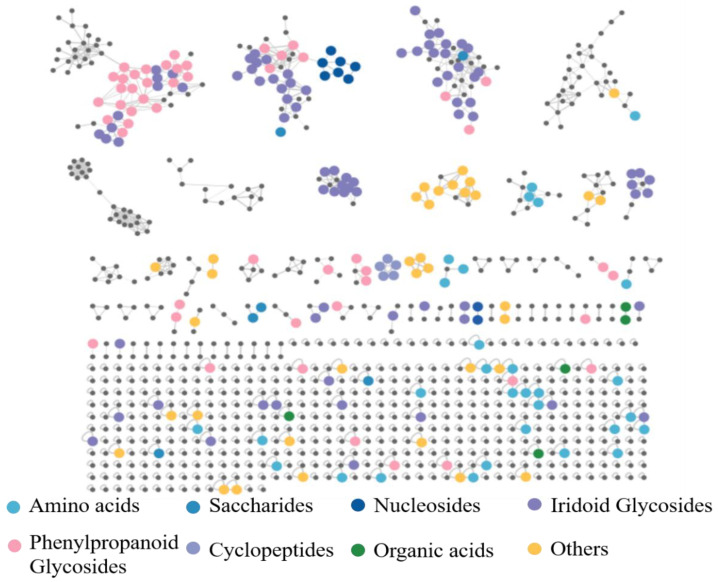
The MN diagram of differently processed SR in both positive ion mode and negative ion mode.

**Figure 2:**
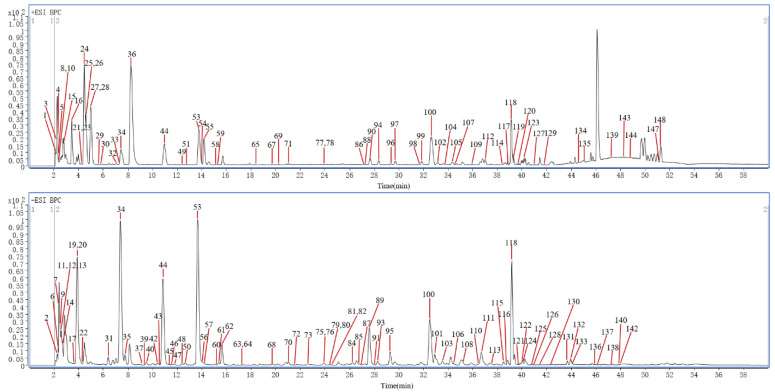
The representative BPC of S1 by UHPLC–Q–TOF–MS in both positive ion mode and negative ion mode.

In this article, a total of 149 compounds were initially identified (Supplementary Figures S1–S8). Among them, 11 chemical compounds were accurately identified by comparing with the corresponding reference substances and 138 chemical compounds were putatively identified with the database comparison and GNPS; the detailed MN diagram can be seen in Figure 1. The base peak chromatograms (BPC) of S1–S6 in both positive ion mode and negative ion mode are shown in Figure 2 and Figures S9–S13. And the detailed mass spectra information of the identified components, including the peak number, retention times (R.T.), compound names, molecular formulas, mass measurement errors (within a ±5 ppm window), adducts, observed molecular weight, fragment ions, and classification, are shown in Supplementary Table S1.

A total of 980 precursor ions contained in the MN, including 30 clusters (nodes ≥ 2) and 407 single nodes, were generated for the differently processed SR in both positive ion mode and negative ion mode; the detailed information is available at https://gnps.ucsd.edu/ProteoSAFe/status.jsp?task=0f50dec1f1274f679935181639590aba (accessed on 3 March 2024). As shown in Figure 1, nucleosides and cyclopeptides could be well aggregated into clusters separately, while iridoid glycosides (IGs) and phenylpropanoid glycosides (PGs) were easily cross–aggregated into one cluster because of the similar structural fragments in their structures, such as glucose, rhamnose, cinnamoyl, feruloyl, and so on.

#### 2.1.1. Identification of Iridoid Glycosides

Iridoids are the dominant secondary metabolites of the genus *Scrophularia*, and structurally they are cyclopentano[c]pyran monoterpenoids with a hemiacetal hydroxyl group; thus, they are usually found in plants known as glycosides [21,22,23]. Typically, IGs generate the [M+Na]^+^ adduct in the positive ion mode, and [M−H]^−^ or [M+HCOO]^−^ adducts in the negative ion mode. Generally, the characteristic neutral losses of IGs were glucose (Glc, 162.0528 Da), H_2_O (18.0106 Da), and CO_2_ (43.9898 Da). If the structures of IGs contain phenyl acryloyl substituent groups such as feruloyl, caffeoyl, *p*–coumaroyl or cinnamoyl, the ester bond will be broken much more easily, and then the glucose and H_2_O will be lost. In addition, the aglycone of IGs may further undergo ring–opening cracking [24].

**Figure 3:**
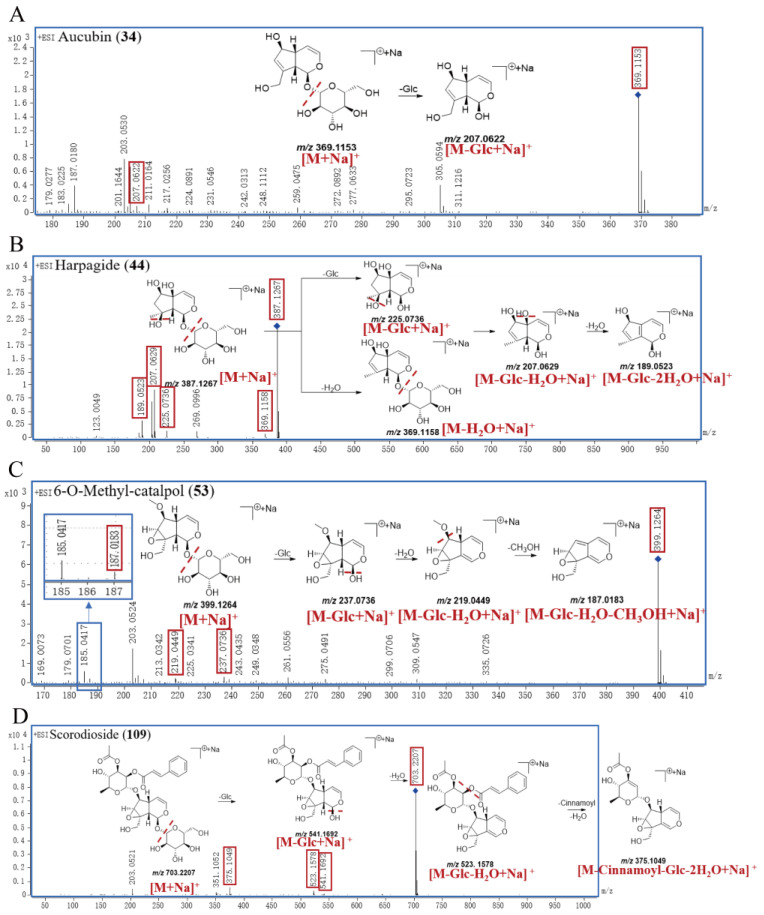
The proposed fragmentation pathway of aucubin (**A**), harpagide (**B**), 6–O–methyl–catalpol (**C**), and scorodioside (**D**) in SR.

A total of 52 IGs were finally identified in this research. Peak **34** generated the [M+Na]^+^ ion at *m/z* 369.1153 and the molecular formula was inferred to be C_15_H_22_O_9_, and then yielded product ions at *m/z* 207.0622 due to the loss of Glc (162.0528 Da). Compared with the self–built database and reference substance, peak **34** was accurately identified as aucubin; the proposed fragmentation pathway of aucubin is shown in Figure 3A. 

Peak **44** exhibited a [M+Na]^+^ ion at *m/z* 387.1267 and the molecular formula was inferred to be C_15_H_24_O_10_. Peak **44** successively generated the product ions at *m/z* 369.1158, 225.0736, 207.0629, and 189.0523, which correspond to [M−H_2_O+Na]^+^, [M−Glc+Na]^+^, [M−Glc−H_2_O+Na]^+^, and [M−Glc−2H_2_O+Na]^+^, respectively. Compared with the self–built database and reference substance, peak **44** was accurately identified as harpagide; the proposed fragmentation pathway is shown in Figure 3B.

Peak **53** showed a [M+Na]^+^ ion at *m/z* 399.1264 with the molecular formula of C_16_H_24_O_10_, and it subsequently produced product ions at *m/z* 237.0736, 219.0449, and 187.0183, corresponding to [M−Glc+Na]^+^, [M−Glc−H_2_O+Na]^+^, and [M−Glc−H_2_O−CH_3_OH+Na]^+^. Compared with the self–built database and reference substance, peak **53** was accurately identified as 6–O–methyl–catalpol; the proposed fragmentation pathway of 6–O–methyl–catalpol is shown in Figure 3C.

The parent ion [M+Na]^+^ of peak **109** was at *m/z* 703.2207, and the molecular formula was inferred to be C_32_H_40_O_16_. Peak **109** successively yielded the characteristic fragment ion at *m/z* 541.1692 [M−Glc+Na]^+^, *m/z* 523.1578 [M−Glc−H_2_O+Na]^+^, and *m/z* 375.1049 [M−Cinnamoyl−Glc−2H_2_O+Na]^+^. Compared with the self–built database, peak **109** was definitively identified as scorodioside, with the proposed fragmentation pathway shown in Figure 3D. 

#### 2.1.2. Identification of Phenylpropanoid Glycosides

PGs, as one of the representative constituents in SR, are structurally characterized by phenylethanol bound to a *β*–(D)–glucopyranoside [25]. In general, based on the number of linked sugars in the structure, PGs can be categorized into monosaccharide glycosides, disaccharide glycosides, and trisaccharide glycosides [10]. By analyzing the structures of PGs, the central sugar that is directly linked to the aglycone is glucose, which is usually directly connected with aglycone in monosaccharide glycosides. Commonly, in addition to monosaccharide glycosides, rhamnose (Rha) or arabinose (Ara) or Glc is attached to the position of C–3 or C–6 of central glucose. However, the C–4 and C–6 positions of the central glucose are often attached to phenyl acryloyl functional groups such as feruloyl, caffeoyl, *p*–coumaroyl, or cinnamoyl [26,27]. Generally, PGs have strong responsiveness in negative ion mode, and often generate the [M−H]^−^ and [M+HCOO]^−^ adducts with the characteristic neutral losses of Glc (162.0528 Da), H_2_O (18.0106 Da), feruloyl (176.0473 Da), caffeoyl (176.0473 Da), *p*–coumaroyl (146.0368 Da), cinnamoyl (130.0419 Da), and CO (27.9949 Da).

**Figure 4:**
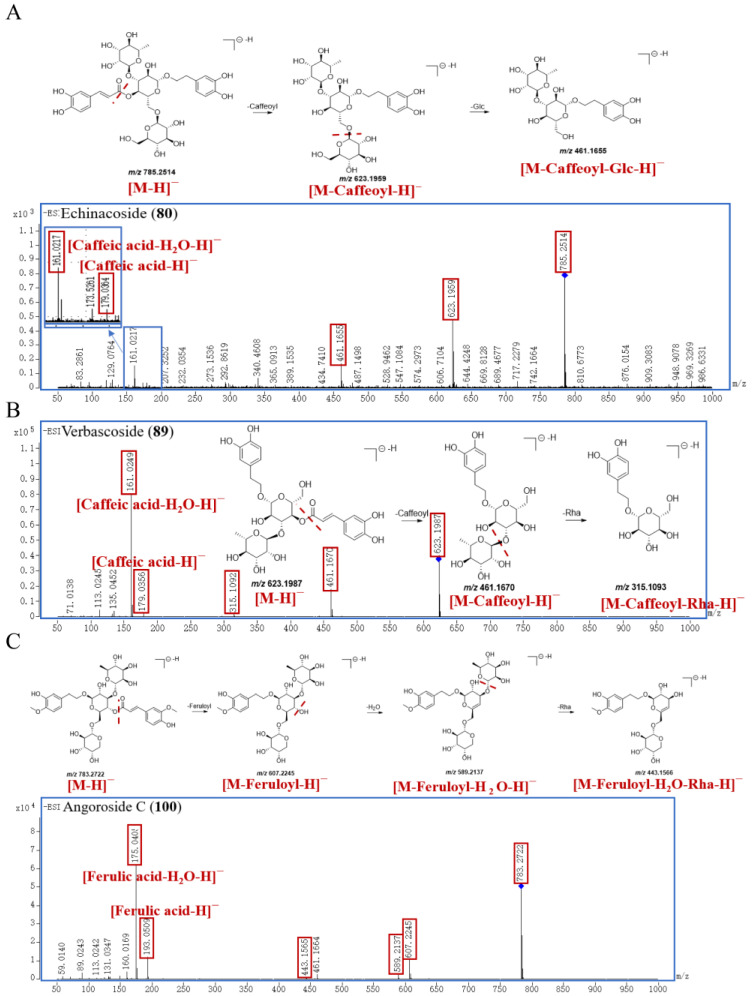
The proposed fragmentation pathway of echinacoside (**A**), verbascoside (**B**), and angoroside C (**C**) in SR.

Statistically, 31 PGs have been identified in this work. Peak **80** generated the [M−H]^−^ ion at *m/z* 785.2514 and the molecular formula was inferred to be C_35_H_46_O_20_. Peak **80** successively yielded product ions at *m/z* 623.1959, 461.1655, 179.0364, and 161.0217, which correspond to [M−Caffeoyl−H]^−^, [M−Caffeoyl−Glc−H]^−^, [Caffeic acid−H]^−^, and [Caffeic acid−H_2_O−H]^−^. Peak **80** was finally identified by comparison with the self–built database as echinacoside, and the proposed fragmentation pathway of echinacoside is shown in Figure 4A.

The parent ion [M−H]^−^ of peak **89** was at *m/z* 623.1987 with the molecular formula of C_29_H_36_O_15_. The characteristic fragment ions of peak **89** are *m/z* 461.1670, 315.1093, 179.0356, and 161.0249, corresponding to [M−Caffeoyl−H]^−^, [M−Caffeoyl−Rha−H]^−^, [Caffeic acid−H]^−^, and [Caffeic acid−H_2_O−H]^−^, respectively. Compared with the self–built database and reference substance, peak **89** was accurately identified as verbascoside; the proposed fragmentation pathway is exhibited in Figure 4B. Peak **95** produced the [M−H]^−^ at 623.1997, and its molecular formula is C_29_H_36_O_15_, too. And peak **95** further cleaved into major characteristic ions at *m/z* 461.1664 [M−Caffeoyl−H]^−^, *m/z* 315.1083 [M−Caffeoyl−Rha−H]^−^, *m/z* 179.0354 [Caffeic acid−H]^−^, and *m/z* 161.0246 [Caffeic acid−H_2_O−H]^−^, whose fragmentation pattern was consistent with that of verbascoside. And peak **95** was identified as an isomer of verbascoside. Compared with the self–built database and reference substance, peak **95** was explicitly identified as isoverbascoside, with a retention time of 29.295 min, which was longer than that of verbascoside (27.590 min).

Peak **100** exhibited a [M−H]^−^ ion at *m/z* 783.2722 and the molecular formula was inferred to be C_36_H_48_O_19_. Peak **100** generated the characteristic product ions at *m/z* 607.2245, 589.2137, 443.1566, 193.0509, and 175.0405, which were related to [M−Feruloyl−H]^−^, [M−Feruloyl−H_2_O−H]^−^, [M−Feruloyl−H_2_O−Rha−H]^−^, [Ferulic acid−H]^−^, and [Ferulic acid−H_2_O−H]^−^, respectively. Compared with the self–built database and reference substance, peak **100** was identified as angoroside C, and the proposed fragmentation pathway is shown in Figure 4C.

#### 2.1.3. Identification of Cyclopeptides

As a unique class of naturally occurring privileged molecules, cyclopeptides are polypeptide chains which are formed by amide bonds in a circular sequence between proteinogenic or nonproteinogenic amino acids [28]. Cyclopeptides have attracted attention in therapeutics, drug design, and pharmacological applications, due to their low toxicity, good binding affinity, structural rigidity, receptor selectivity, and biochemical stability [28,29,30]. Normally, cyclopeptides have strong mass spectrum signals in positive ion mode, and often form the [M+H]^+^ adduct ion peak with the common neutral losses of H_2_O (18.0106 Da) and aminoacyl. In this study, five leucine or isoleucine cyclopeptides were identified, which were peak **78**, **86**, **97**, **99**, and **104**. And the common neutral loss in the fragmentation process was H_2_O (18.0106 Da), and leucyl or isoleucyl (113.0841 Da).

**Figure 5:**
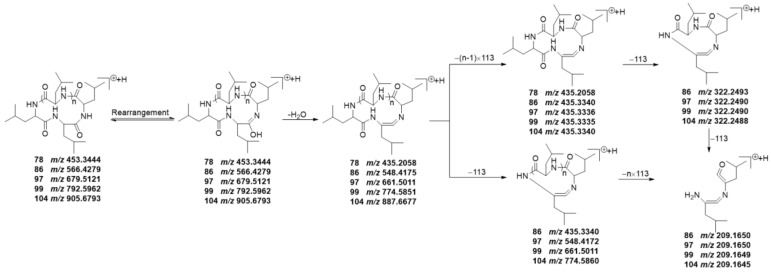
The proposed mass spectrometry cleavage pathways of cyclopeptides.

**Figure 6:**
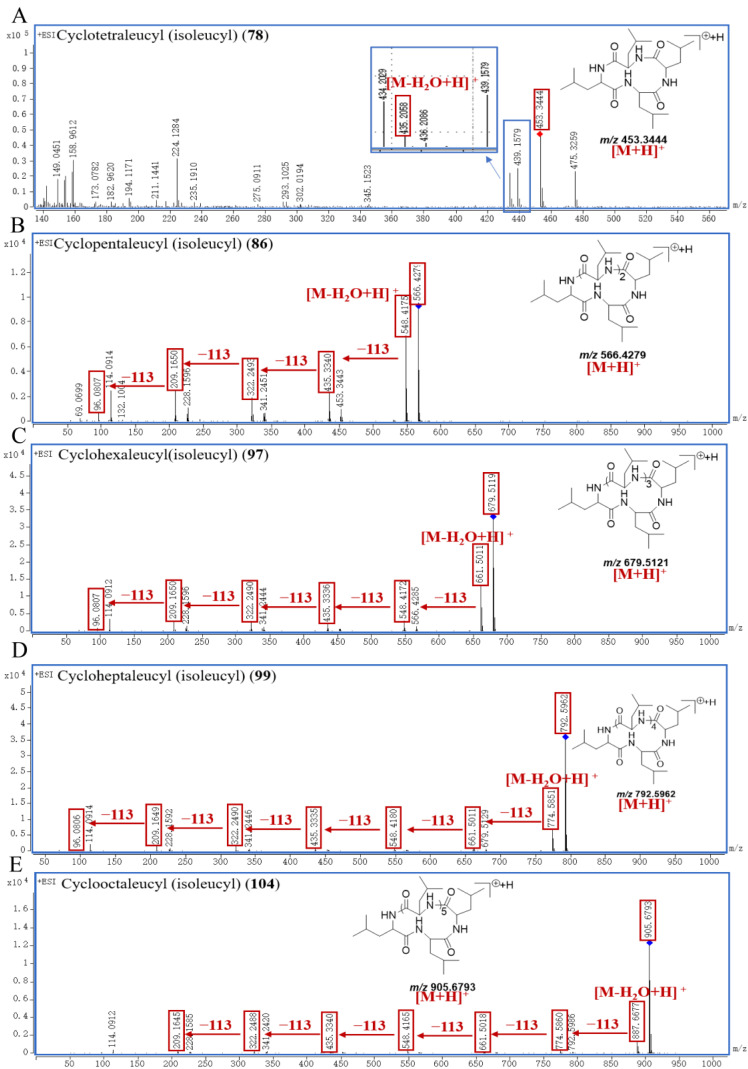
The specific fragmentation pathways of cyclotetraleucyl (isoleucyl) (**A**), cyclopentaleucyl (isoleucyl) (**B**), cyclohexaleucyl (isoleucyl) (**C**), cycloheptaleucyl (isoleucyl) (**D**), and cyclooctaleucyl (isoleucyl) (**E**) in SR.

Peak **78** generated a [M+H]^+^ ion at *m/z* 453.3444, and its molecular formula was supposed to be C_24_H_44_N_4_O_4_; peak **86** generated a [M+H]^+^ ion at *m/z* 566.4279 with its molecular formula of C_30_H_55_N_5_O_5_; peak **97** showed a [M+H]^+^ ion at *m/z* 679.5119, and its molecular formula was inferred to be C_36_H_66_N_6_O_6_; peak **99** exhibited a [M+H]^+^ ion at *m/z* 792.5962, and its molecular formula was inferred to be C_42_H_77_N_7_O_7_; the parent ion [M+H]^+^ of compound peak **104** was at *m/z* 905.6793 with the molecular formula of C_48_H_88_N_8_O_8_. According to the fragmentation patterns in the literature [31], peak **78** was finally identified as cyclotetraleucyl (isoleucyl), peak **86** was finally identified as cyclopentaleucyl (isoleucyl), peak **97** was finally identified as cyclohexaleucyl (isoleucyl), peak **99** was finally identified as cycloheptaleucyl (isoleucyl), and peak **104** was finally identified as cyclooctaleucyl (isoleucyl). The proposed mass spectrometry cleavage pathways of cyclopeptides are shown in Figure 5, and the specific fragmentation pathways of the peaks **78**, **86**, **97**, **99**, and **104** spectra are shown in Figure 6.

#### 2.1.4. Identification of New Compounds

**Figure 7:**
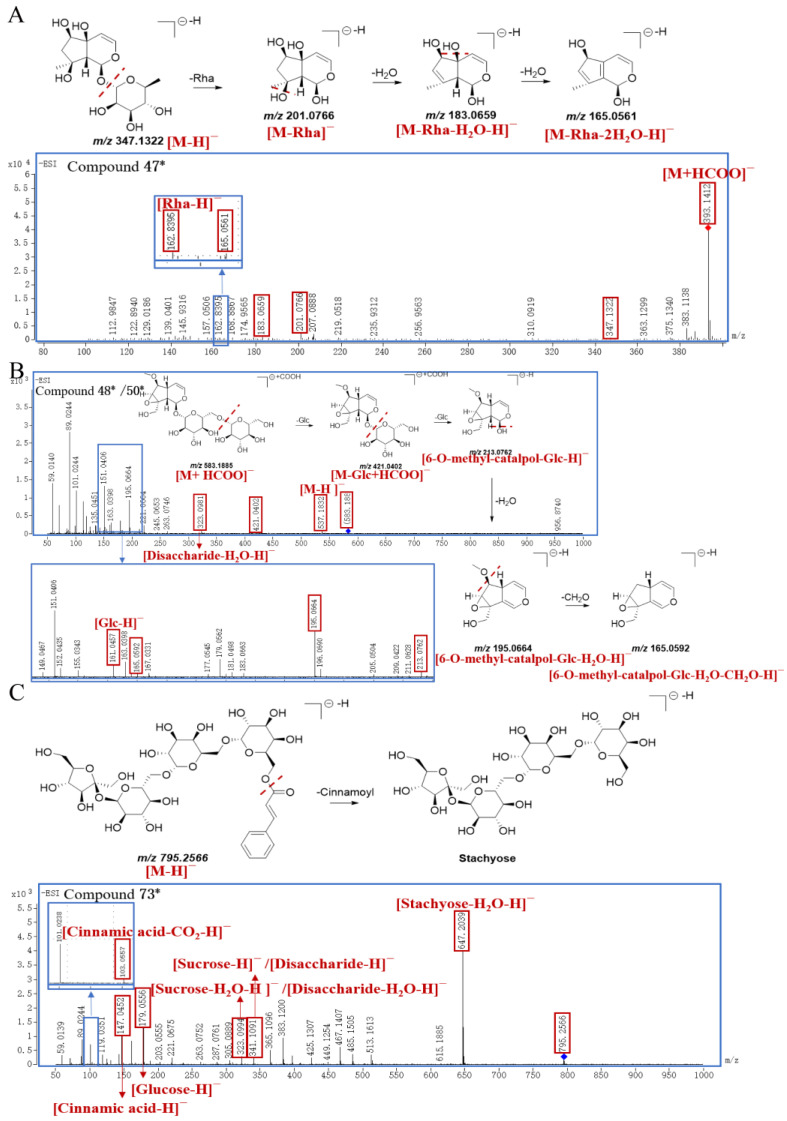
The proposed fragmentation pathway of compound 47* (**A**), compound 48*/50* (**B**), and compound 73* (**C**) in SR.

In this study, four new compounds were identified (three of them are IGs, and one of them is a PG). Peak 47* showed a [M+HCOO]^−^ ion at *m/z* 393.1412 and a [M−H]^−^ ion at *m/z* 347.1322, and the molecular formula was inferred to be C_15_H_24_O_9_. Then, peak 47* further cleaved into *m/z* 201.0766, 183.0659, 165.0561, and 162.8395, which correspond to [M−Rha]^−^, [M−Rha−H_2_O−H]^−^, [M−Rha−2H_2_O−H]^−^, and [Rha−H]^−^, respectively. Based on structural deduction, peak 47* was tentatively identified as *α–L*–rhamnopyranoside, (1*S*,4a*S*,5*R*,7*S*,7a*R*)–1,4a,5,6,7,7a–hexahydro–4a,5,7–trihydroxy–7–methylcyclopenta[c]pyran–1–yl(9CI,ACI); proposed fragmentation pathway is shown in Figure 7A.

Peak 48^*^ generated a [M+HCOO]^−^ ion at *m/z* 583.1885 and a [M−H]^−^ ion at *m/z* 537.1832 with the molecular formula of C_22_H_34_O_15_. And peak 48* produced characteristic ions at *m/z* 421.0402 [M−Glc+HCOO]^−^, *m/z* 323.0981 [Disaccharide−H_2_O−H]^−^, *m/z* 213.0762 [6−O−methyl−catalpol−Glc−H]^−^, *m/z* 195.0664 [6−O−methyl−catalpol−Glc−H_2_O−H]^−^, *m/z* 165.0592 [6−O−methyl−catalpol−Glc−H_2_O−CH_2_O−H]^−^, and *m/z* 161.0457 [Glc−H]^−^, respectively. Through structural deduction, peak 48* was tentatively identified as (2*R*,3*S*,4*S*,5*R*,6*R*)–2–(hydroxymethyl)–6–(((2*R*,3*S*,4*S*,5*R*,6*S*)–3,4,5–trihydroxy–6–(((1a*S*,1b*S*,2*S*,5a*R*,6*S*,6a*S*)–1a(hydroxymethyl)–6–methoxy–1a,1b,2,5a,6,6a–hexahydrooxireno [2′,3′:4,5]cyclopenta[1,2–c]pyran–2–yl) oxy)tetrahydro–2H–pyran–2–yl)methoxy)tetrahydro–2H–pyran–3,4,5–triol, and the proposed fragmentation pathway is shown in Figure 7B. Similarly, the fragmentation pattern of peak 50* was consistent with that of peak 48*, and peak 50* was presumed as an isomer of peak 48*.

The parent ion [M−H]^−^ of peak 73* was at *m/z* 795.2566, and the molecular formula was inferred to be C_33_H_48_O_22_. The characteristic fragment ions of peak 73^*^ were *m/z* 647.2039 [Stachyose−H_2_O−H]^−^, *m/z* 341.1091 [Sucrose−H]^−^ or [Disaccharide−H]^−^, *m/z* 323.0994 [Sucrose−H_2_O−H]^−^ or [Disaccharide−H_2_O−H]^−^, *m/z* 179.0556 [Glucose−H]^−^, *m/z* 147.0452 [Cinnamic acid−H]^−^, and *m/z* 103.0557 [Cinnamic acid−CO_2_−H]^−^, respectively. Due to the widespread presence of endophytic fungi in *S. ningpoensis*, it was speculated that the regioselective chemical acylation of stachyose with cinnamic acid might be catalyzed by some lipases towards the 6–OH of the terminal galactose [32]. Based on the above analysis, peak 73^*^ was tentatively identified as ((2*R*,3*R*,4*S*,5*R*,6*S*)–6–(((2*R*,3*R*,4*S*,5*R*,6*S*)–6–(((2*R*,3*S*,4*S*,5*R*,6*R*)–6–(((2*S*,3*S*,4*S*,5*R*)–3,4–dihydroxy–2,5–bis(hydroxymethyl)tetrahydrofuran–2–yl)oxy)–3,4,5–trihydroxytetrahydro–2H–pyran–2–yl)methoxy)–3,4,5–trihydroxytetrahydro–2H–pyran–2–yl)methoxy)–3,4,5–trihydroxytetrahydro–2H–pyran–2–yl)methyl cinnamate; the proposed fragmentation pathway is exhibited in Figure 7C.

### 2.2. Analysis of Changes in Compounds in Differently Processed SR

**Figure 8:**
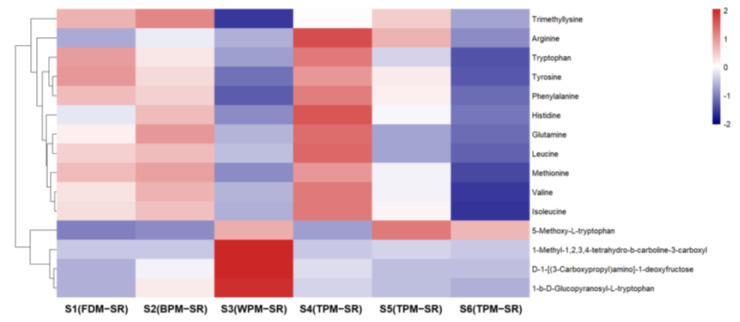
The heatmap of amino acids in differently processed SR.

**Figure 9:**
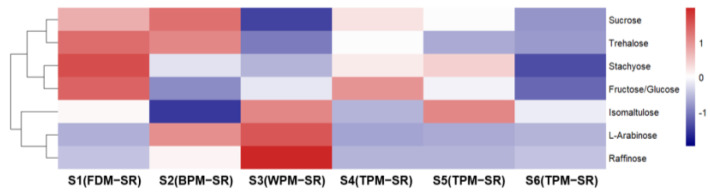
The heatmap of saccharides in differently processed SR.

**Figure 10:**
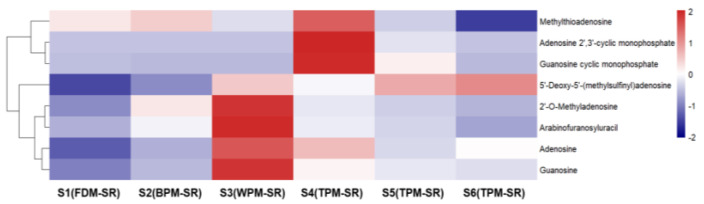
The heatmap of nucleosides in differently processed SR.

**Figure 11:**
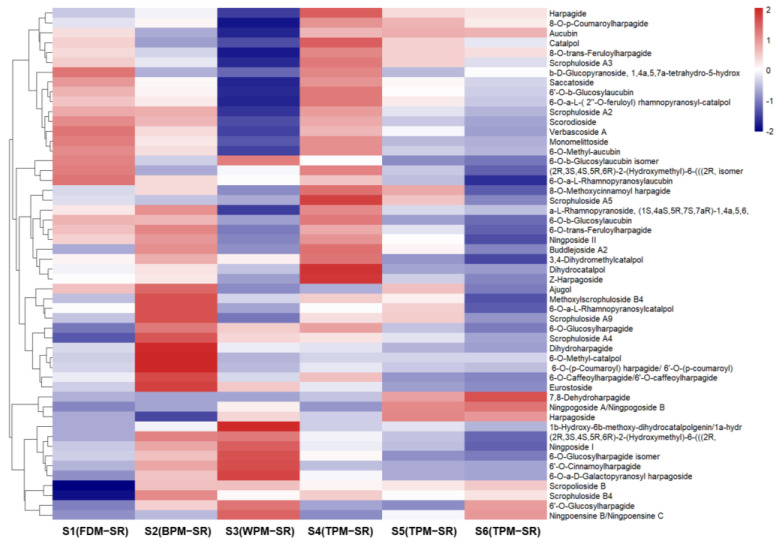
The heatmap of iridoid glycosides in differently processed SR.

**Figure 12:**
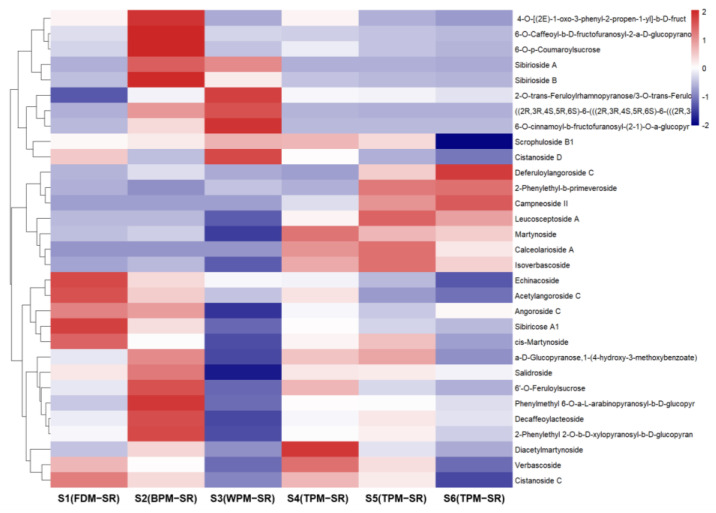
The heatmap of phenylpropanoid glycosides in differently processed SR.

**Figure 13:**
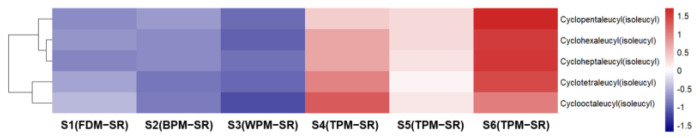
The heatmap of cyclopeptides in differently processed SR.

**Figure 14:**
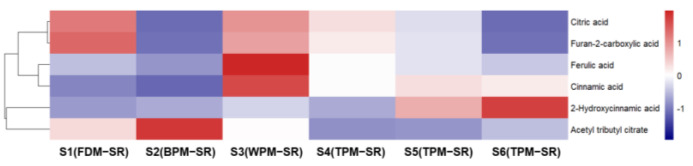
The heatmap of organic acids in differently processed SR.

**Figure 15:**
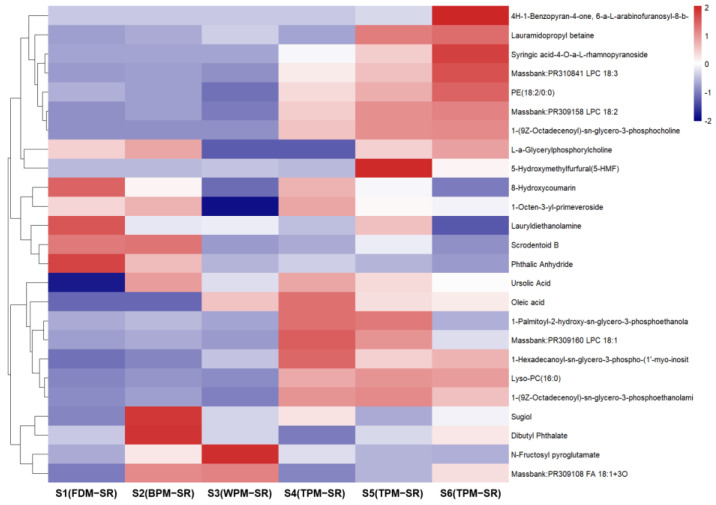
The heatmap of other components in differently processed SR.

Figure 8, Figure 9, Figure 10, Figure 11, Figure 12, Figure 13, Figure 14 and Figure 15 are the heatmaps of amino acids, saccharides, nucleosides, IGs, PGs, cyclopeptides, organic acids, and other components in the differently processed samples of SR (the names of compounds in the figures are ≤50 bytes). The differently processed samples S1, S2, S3, S4, S5 and S6 of SR are treated by the same preparation method, so the relative content of the same compound can be determined by comparing the ion abundance of the same compound in the mass spectrometry data of different samples.

The relative contents of histidine (**2**), arginine (**3**), glutamine (**4**), valine (**15**), methionine (**16**), tyrosine (**24**), isoleucine (**25**), leucine (**27**), phenylalanine (**36**), and tryptophan (**55**) are the highest in 1h–TPM–SR, and the content of free amino acids in the FDM–SR and BPM–SR were second only to that of the TPM–SR, but gradually decreased with the prolongation of the steaming time. It was suggested that the short–time steaming process will promote the production of free amino acids, but a long–time heating will significantly reduce the content of free amino acids, which may be related to the Maillard reaction [33]. Furthermore, some amino acids may be converted into other components during the steaming process; for example, leucine or isoleucine in the SR might be converted into cyclopeptides during the high temperature steaming process (Figure 8), with the content of the cyclopeptides in the S6 sample steamed for 24 h being the highest. The relative contents of D–1–[(3–carboxypropyl) amino]–1–deoxyfructose (**8**), 1–*β*–D–glucopyranosyl–L–tryptophan (**52**) and 1–methyl–1,2,3,4–tetrahydro–*β*–carboline–3–carboxylic acid (**66**) in WPM–SR are the highest, which were speculated to arise from the Maillard reaction and enzymatic reactions during the sweating process [34,35].

An observation of Figure 9 shows that FDM and BPM can avoid decreasing the relative contents of oligosaccharides (**9**, **11**, and **13**) to a certain extent in SR. With the extension of the steaming time, the relative content of oligosaccharides in SR decreased obviously, but the relative content of monosaccharides did not significantly increase, which indicates that the TPM may promote the conversion of oligosaccharides into non–monosaccharide components. In addition, the WPM not only could promote the hydrolysis of oligosaccharides or glycosides but could also promote the isomerization of sucrose (**11**) to form isomaltulose (**6**).

From Figure 10, it can be found that the relative content of nucleosides in FDM–SR and BPM–SR is lower, but the WPM–SR and 1h–TPM–SR contained relatively higher nucleosides. Additionally, the structures of the nucleosides with a higher content in WPM–SR are mainly dominated by basal nucleosides (**10**, **28**, **29**). The short–time steaming process was beneficial for the generation of methylthioadenosine (**54**), adenosine 2′,3′–cyclic monophosphate (**18**), and guanosine cyclic monophosphate (cGMP, **21**). cGMP and cyclic adenosine monophosphate (cAMP), as second messengers in cell signal transduction, can exert their physiological effects through bidirectional regulation [36]. Evidence suggests that an increase in the cAMP/cGMP ratio in the body corresponds to yin deficiency [37], which could be regulated by cGMP in 1h–TPM–SR.

As can be seen from Figure 11, the FDM, BPM and short–time TPM can protect the IGs well, including the glycosyl and phenyl acryloyl substituent groups in their structures, such as feruloyl, caffeoyl, *p*–coumaroyl or cinnamoyl. In addition, long–time TPM gradually reduced the relative content of IGs in SR, especially in S6, so it is speculated that the glycosidic bonds and ester bonds of IGs might undergo high–temperature degradation during the process of prolonged steaming. During the above processing, the relative content of 7,8–dehydroharpagide (**30**) continuously increased, accompanied by a decrease in the relative content of harpagide (**44**). In addition, compared with FDM, BPM, and short–time TPM, WPM mainly increased the relative content of harpagide derivatives (**46**, **114**, and **117**), which was probably produced by the glycosylation of harpagide and harpagoside. And the significant increase in relative content of **87** in WPM–SR may be attributed to the enzymatic hydrolysis reaction of 6–O–methyl–catalpol (**53**).

Observing Figure 12, it can be seen that the relative content of monosaccharide glycosides and disaccharide glycosides of PGs are higher in BPM–SR, among which, the relative content of the sucrose phenylpropionic acid ester (**64**, **67**, **70**, **71**, **91**, and **96**) was remarkably higher than the other processed SR. In the FDM–SR and TPM–SR, the relatively abundant PGs were mainly disaccharide glycosides and trisaccharide glycosides. And WPM significantly reduces the content of the relatively abundant components in the FDM–SR, such as echinacoside (**80**), angoroside C (**100**), sibiricose A1 (**63**), et al. So, it is speculated that enzymatic reaction might induce the hydrolysis or ester of several PGs and oligosaccharides, which is similar to that of the IGs. Therefore, based on the change rules of the IGs and PGs in differently processed SR, it is known that WPM and long–time steaming will reduce SR’s relative contents of PGs, which is probably related to the enzymatic reaction or high–temperature degradation during the process of sweating and long–time steaming. Furthermore, in WPM–SR, stachyose (**9**) could be acylated under thermolysin catalysis [38] or subtilisin catalysis [39] to form the new compound **73**.

By observing Figure 13, it can be seen that the relative contents of cyclopeptides (**78**, **86**, **97**, **99**, and **104**) were all lower in SR processed by FDM, BPM or WPM than that of TPM–SR. Moreover, the relative contents of cyclopeptides in SR treated by steaming for 24 h is the highest. This is the first report on the cyclopeptides of SR and their changes under different processing methods. Combining the change rules of leucine and isoleucine content in Figure 8, it is speculated that leucine and isoleucine might be gradually dehydrated and condensed to generate cyclopeptides during the long–time and high–temperature steaming process, which indicates that cyclotetraleucyl (isoleucyl) (**78**), cyclopentaleucyl (isoleucyl) (**86**), cyclohexaleucyl (isoleucyl) (**97**), cycloheptaleucyl (isoleucyl) (**99**), and cyclooctaleucyl (isoleucyl) (**104**) might be the potential Q–markers of the TPM–SR.

It can be seen from Figure 14 that the relative contents of citric acid (**19**) and furan–2–carboxylic acid (**20**) were the highest in FDM–SR, and the relative contents of cinnamic acid (**124**) and ferulic acid (**85**) were the highest in WPM–SR. Furthermore, the relative content of 2–hydroxycinnamic acid (**81**) could gradually increase with the extension of steaming time in the TPM–SR, while the relative contents of organic acids in BPM–SR is the lowest. Based on the change rules of IGs and PGs, it is suggested that the relatively higher content of cinnamic acid and ferulic acid in WPM–SR may be related to the hydrolysis of cinnamoylated or feruloylated IGs or PGs.

From Figure 15, it can be found that the relative content of lipids is lower in the SR samples processed by FDM, BPM, and WPM, while the relative content of lipids is higher in the TPM–SR, such as massbank–PR310841 LPC 18:3 (**134**), PE (18:2/0:0) (**136**), and massbank–PR309158 LPC 18:2 (**137**). The contents of 8–hydroxycoumarin (**90**), lauramidopropyl betaine (**129**), and phthalic anhydride (**148**) were higher in the FDM–SR. And the contents of sugiol (**144**), scrodentoid B (**143**), and dibutyl phthalate (**147**) were higher in the BPM–SR. WPM could cause an increase in the contents of N–fructosyl pyroglutamate (**17**) and massbank–PR309108 FA 18:1+3O (**126**). The short–time TPM and BPM could avoid the excessive generation of genotoxic 5–hydroxymethylfurfural, which has been found to possess mutagenic and DNA strand–breaking activity [40].

## 3. Materials and Methods

### 3.1. Chemicals and Reagents

The analytical standard of L–tyrosine was procured from the National Institutes for Food and Drug Control (Beijing, China); aucubin, L–phenylalanine, harpagide, L–tryptophan, verbascoside, isoverbascoside, angoroside C, harpagoside, and cinnamic acid were procured from Shanghai Standard Technology Co., Ltd. (Shanghai, China); and 6–O–methyl–catalpol was isolated and purified from the fresh roots of *S. ningpoensis*. The purity of all the reference standards was greater than 98% by HPLC. The HPLC–grade acetonitrile and methanol, as well as the HPLC–grade phosphoric acid, were procured from Shanghai Titan Scientific Co., Ltd. (Shanghai, China). Distilled water was obtained using a Milli–Q system (Millipore, Bedford, MA, USA). Other solvents were of analytical grade (Sino Pharm Chemical Reagent, Shanghai, China).

### 3.2. Sample Preparation

The fresh roots of *S. ningpoensis* (FRSN) were purchased from Pan’an in December 2019, Zhejiang, and authenticated by Professor Yiming Li (School of Pharmacy, Shanghai University of Traditional Chinese Medicine). All voucher specimens were deposited at the School of Pharmacy, Shanghai University of Traditional Chinese Medicine, China. All the samples were processed in our laboratory. S1 was processed through the “freeze–drying” method (FDM); S2 was processed by BPM, which involved oven–drying the slices of FRSNs at 55 °C until it is completely dry; S3 was processed by the WPM which involved oven–drying the FRSNs at 55 °C for 1 day first, and then allowing the samples to semidry and rest inside a black bag for 2 days; these steps were repeated twice, and the the WPM–SR was finally placed –in the oven at 55℃ until it was completely dry; S4–S6 were processed by the TPM, which involved steaming the FRSNs in a steamer, taking them out at regular intervals (1 h, 12 h, and 24 h), and finally oven–drying the TPM–SR at 55℃ until it was completely dry.

The dried samples were powdered and passed through a 50 mesh sieve. Each sample (0.5 g) was accurately weighed and transferred to a 100 mL conical flask with a stopper. Ten milliliters of 50% methanol (*v*/*v*) was added and the flask was weighed again. The mixture was subjected to ultrasonication for 45 min to obtain an extract, which was then cooled to room temperature and brought to its original weight with 50% methanol. Finally, the supernatant solution was filtered using a 0.22 µm membrane before analysis.

### 3.3. UPLC–Q–TOF–MS Analysis

The mass spectrometry detection was performed on a 1290 HPLC system and a 6545 ultrahigh–definition quadrupole time–of–flight mass spectrometer (Q–TOF–MS; Agilent Technologies, Santa Clara, CA, USA) with an Agilent Extend–C_18_ column (4.6 mm × 250 mm, 5 µm). The mobile phase was composed of acetonitrile (A) and 0.1% formic acid in water (B) (*v*/*v*). And the gradient elution progressed as follows: 0–8 min, 3–6 %A; 8–18 min, 6–15 %A; 18–25 min, 15–20 %A; 25–35 min, 20–25 %A; 35–38 min, 25–47 %A; 38–45 min, 47–75 %A; 45–50 min, 75–80 %A. The flow rate was 1.0 mL/min, and the sample injection volume was 2 µL.

The sample solution was detected through Q–TOF–MS’s data–dependent acquisition (DDA) mode with the electrospray ionization (ESI) source in both the positive ion mode and negative ion mode. In the DDA mode, the five most-abundant ions were selected in the first stage and then crushed and analyzed in the second stage, and these data-dependent-type data are used for the establishment of MN. The operational parameters of ESI-MS were as follows: the capillary voltage was ±4 kV; fragmentor, 120 V; nozzle voltage, 500 V. The scan range was *m/z* 100~1200. Nitrogen was used as the sheath gas at 12 L/min and 350 °C, the nebulizer gas at 35 psi, and the drying gas at 12 L/min and 350 °C. And before measurements, the Q-TOF-MS was calibrated externally using a series of homogeneously substituted fluorinated triazatriphosphorines (*m/z* 50–3200) to ensure a mass accuracy of less than 2 ppm and a mass resolution of 20000 (*m/z* 322.0487).

### 3.4. Establishment of the Chemical Compounds Database

A self-built database of compounds from *S. ningpoensis* was established by searching the literature on the PubMed, SciFinder, Web of Science, and Chinese National Knowledge Infrastructure databases. Consequently, 385 compounds, including their names, chemical structures, formulas, molecular weights, and characteristic fragments, were discovered, which were able to provide reference information for better identifying and screening the chemical constituents of *S. ningpoensis*.

### 3.5. Data Analysis of the Molecular Networking in the GNPS Platform and Heatmap Diagrams

The acquired MS/MS spectral data files were converted into “32-bit”.mzXML format by using the MSConvert software (version 3.0.22116). To manipulate MS data files in GNPS, the converted files were uploaded to MassIVE, an online public repository for mass spectrometry datasets hosted by the UCSD Center for Computational Mass Spectrometry [15], via WinSCP software (version 5.19.2). And once the data files were uploaded as datasets into GNPS-MassIVE, they were made available for analysis workflows within GNPS. For the molecular network parameters, the values of “Precursor Ion Mass Tolerance” and “Fragment Ion Mass Tolerance” were set to 0.02 Da, respectively. The cosine score was set to be above 0.7 with a minimum of six matched fragment ions. The resulting molecular networks were downloaded and further visualized with the Cytoscape software (version 3.9.1).

The heatmap diagrams were obtained using R Studio software (version 2023.12.0+369) with the “pheatmap” package.

## 4. Conclusions

In this study, the comprehensive chemical constituents from the differently processed roots of *S. ningpoensis* were rapidly identified and systematically analyzed by combining UPLC-Q-TOF-MS coupled with the GNPS data platform. A total of 149 compounds were definitively or tentatively identified through matching their accurate mass signals, suggested molecular formulae, and MS/MS analysis with previously reported data, including 52 IGs, 31 PGs, 15 amino acids, 8 nucleosides, 7 saccharides, 6 organic acids, 5 cyclopeptides, and 25 other components. Notably, with the help of the GNPS data platform and MS^2^ fragment ions, the possible structures of four new compounds (**47**, **48**, **50**, and **73**) were deduced in the differently processed roots of *S. ningpoensis*. Furthermore, as shown in the heatmaps, it was found that WPM as well as long-time TPM could reduce the relative contents of the main IGs and PGs in SR, possibly because the glycosidic bonds and acetyl groups in their structures were hydrolyzed by enzymes during the WPM or decomposed at a high temperature during the long-time TPM. So, short-time steaming can effectively prevent their degradation by inactivating enzymes and preserving glycosides. Additionally, five cyclopeptides [cyclotetraleucyl (isoleucyl) (**78**), cyclopentaleucyl (isoleucyl) (**86**), cyclohexaleucyl (isoleucyl) (**97**), cycloheptaleucyl (isoleucyl) (**99**), and cyclooctaleucyl (isoleucyl) (**104**)] were identified in differently processed roots of *S. ningpoensis* for the first time, and the heatmap results indicated that leucine or isoleucine might be converted to cyclopeptides under the prolonged high-temperature conditions. Furthermore, these results suggest that the processing methods of BPM as well as short-time TPM can effectively protect the effective components such as sucrose (**11**), aucubin (**34**), harpagide (**44**), methylthioadenosine (**54**), and 6-O-*p*-coumaroylsucrosein (**67**) in SR, thus improving the scientific and effectiveness of SR clinical use.

This research provides an advanced strategy to rapidly screen and comprehensively analyze the chemical constituents in raw and processed roots of *S. ningpoensis*, and also reveals the change rules in the relative content of their intrinsic chemical constituents under different processing methods. Additionally, this work lays a foundation for further research on their pharmacological activity, spectrum–effect relationship, and quality control, and provides a useful strategy for further optimizing the industrial processing methods of the roots of *S. ningpoensis*.

## Data Availability

Data are contained within the article or Supplementary Materials.

## Supplementary Materials

The following supporting information can be downloaded at https://www.mdpi.com/article/10.3390/molecules29204866/s1. Figure S1: The 15 identified amino acids in *S. ningpoensis*; Figure S2: The 7 identified saccharides in *S. ningpoensis*; Figure S3: The 8 identified nucleosides in *S. ningpoensis*; Figure S4: The 52 identified iridoid glycosides in *S. ningpoensis*; Figure S5: The 31 identified phenylpropanoid glycosides in *S. ningpoensis*; Figure S6: The 5 identified cyclopeptides in *S. ningpoensis*; Figure S7: The 6 identified organic acids in *S. ningpoensis*; Figure S8: The 25 identified other compounds in *S. ningpoensis*; Figure S9: The BPC of S2 by UHPLC-Q-TOF-MS in both positive ion mode and negative ion mode; Figure S10: The BPC of S3 by UHPLC-Q-TOF-MS in both positive ion mode and negative ion mode; Figure S11: The BPC of S4 by UHPLC-Q-TOF-MS in both positive ion mode and negative ion mode; Figure S12: The BPC of S5 by UHPLC-Q-TOF-MS in both positive ion mode and negative ion mode; Figure S13: The BPC of S6 by UHPLC-Q-TOF-MS in both positive ion mode and negative ion mode; Table S1: Classification and Identification of the metabolites of SR by UHPLC-Q-TOF-MS.

## Abbreviations

BPM, baking processing method; cAMP, cyclic adenosine monophosphate; cGMP, guanosine cyclic monophosphate; FDM, freeze–drying method; GNPS, global natural product social molecular networking; IGs, iridoid glycosides; MN, molecular networking; NPs, natural products; PGs, phenylpropanoid glycosides; R.T., retention times; *S. ningpoensis*, *Scrophularia ningpoensis* Hemsl; SR, Scrophulariae Radix; TCM, traditional Chinese medicine; TPM, steaming processing method; UPLC–Q–TOF–MS, ultra–high performance liquid chromatography coupled with quadrupole time–of–flight mass spectrometry; WPM, sweating processing method.
