# Supplementary material for: The Comprehensive Profiling of the Chemical Components in the Raw and Processed Roots of Scrophularia ningpoensis by Combining UPLC-Q-TOF-MS Coupled with MS/MS-Based Molecular Networking
Source: Molecules. 2024 Oct 14;29(20):4866. doi: 10.3390/molecules29204866 (PMC11510058; doi:10.3390/molecules29204866)
Supplement: Supplementary file 1 [file molecules-29-04866-s001.zip › molecules-3152862-supplementary.pdf]

Supplementary Materials

The Comprehensive Profiling of the Chemical Components in the Raw and Processed Roots of *Scrophularia ningpoensis* by Combining UPLC-Q-TOF-MS Coupled with MS/MS-Based Molecular Networking

Mina Zhang [1], Kaixian Chen [1], Chenguo Feng [2], Fang Zhang [2,*], Liuqiang Zhang [1,*] and Yiming Li [1]

[1] School of Pharmacy, Shanghai University of Traditional Chinese Medicine, Shanghai 201203, China; 0022019058@shutcm.edu.cn (M.Z.); kxchen@simm.ac.cn (K.C.); ymli@shutcm.edu.cn (Y.L.)
[2] The Research Centre of Chiral Drugs, Innovation Research Institute of Traditional Chinese Medicine, Shanghai University of Traditional Chinese Medicine, Shanghai 201203, China; fengcg@shutcm.edu.cn (C.F.)
* Correspondence: fzhang@shutcm.edu.cn (F.Z.); lqzhang@shutcm.edu.cn (L.Z.)

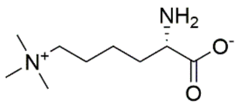

1 Trimethyllysine

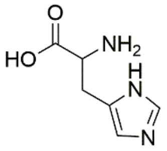

2 Histidine

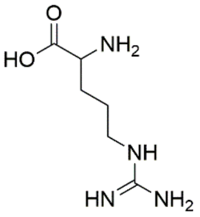

3 Arginine

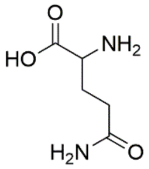

4 Glutamine

8 *D*-1-[(3-Carboxypropyl)amino]-1-deoxyfructose

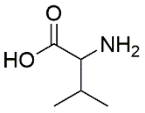

15 Valine

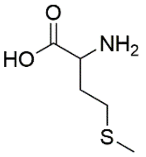

16 Methionine

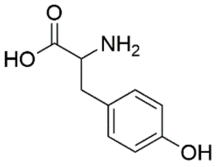

24 Tyrosine

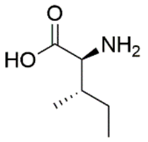

25 Isoleucine

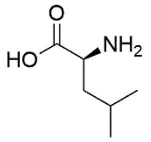

27 Leucine

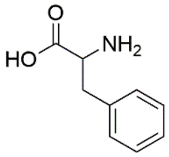

36 Phenylalanine

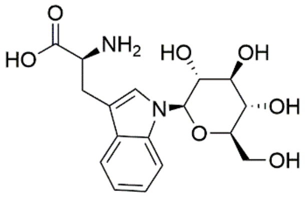

52 1-*β*-*D*-Glucopyranosyl-*L*-tryptophan

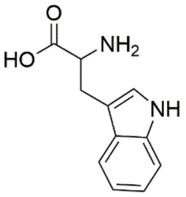

55 Tryptophan

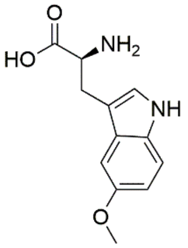

65 5-Methoxy-*L*-tryptophan

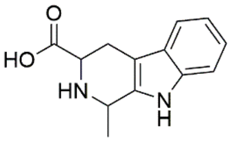

66 1-Methyl-1,2,3,4-tetrahydro-*β*-carboline-3-carboxylic acid

Figure S1. The 15 identified amino acids in *S. ningpoensis.*

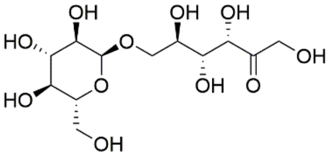

6 Isomaltulose

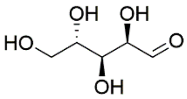

7 *L*-Arabinose

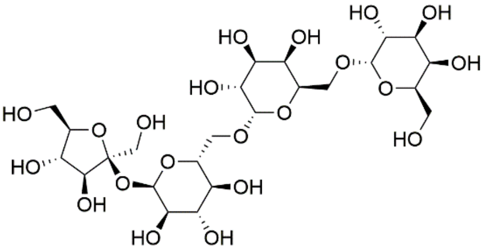

9 Stachyose

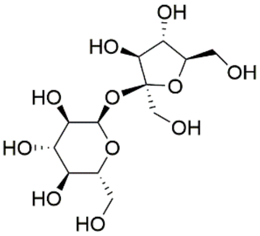

11 Sucrose

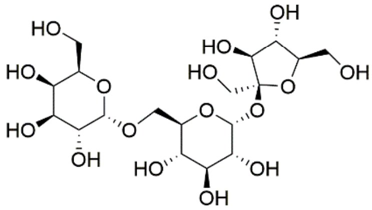

12 Raffinose

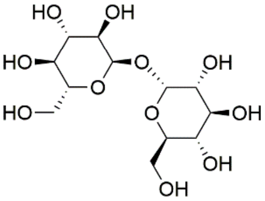

13 Trehalose

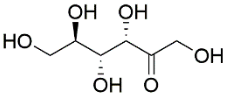

Fructose

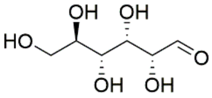

Glucose

14

Figure S2. The 7 identified saccharides in *S. ningpoensis.*

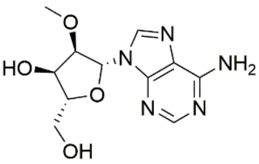

10 2'-O-Methyladenosine

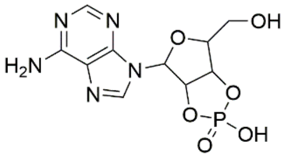

18 Adenosine 2',3'-cyclic monophosphate

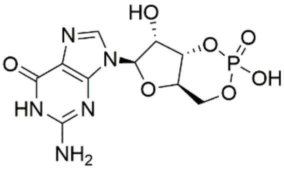

21 Guanosine cyclic monophosphate

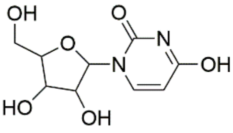

22 Arabinofuranosyluracil

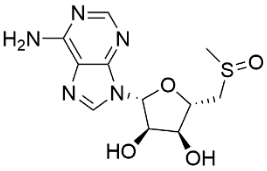

23 5'-Deoxy-5'-(methylsulfinyl)adenosine

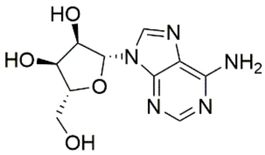

28 Adenosine

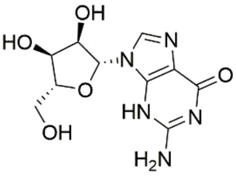

29 Guanosine

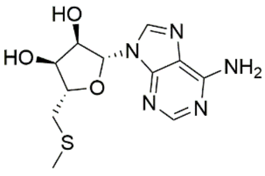

54 Methylthioadenosine

Figure S3. The 8 identified nucleosides in *S. ningpoensis.*

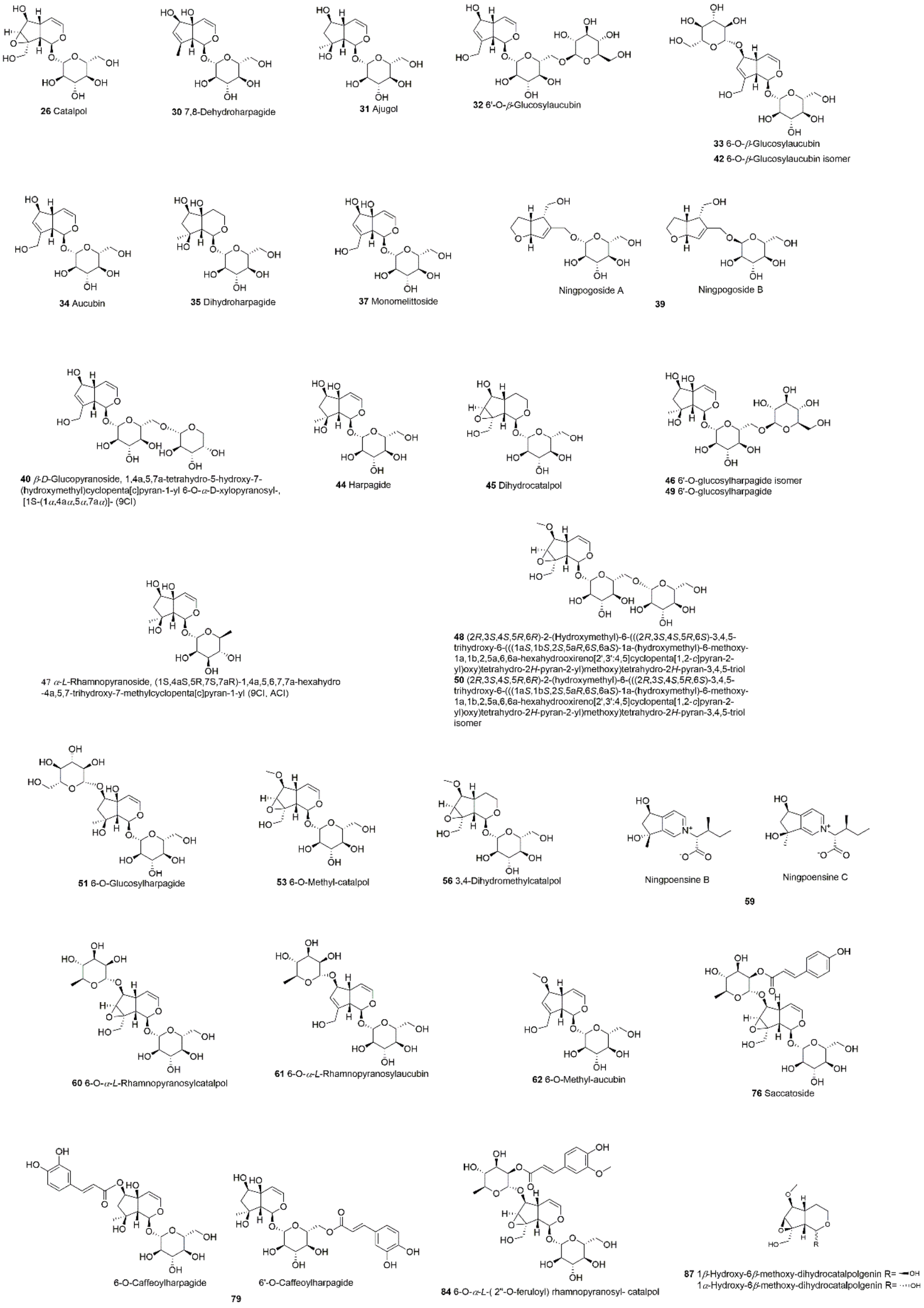

Figure S4. The 52 identified iridoid glycosides in *S. ningpoensis*.

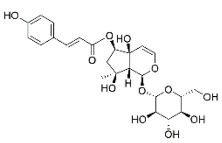

6-O-(*p*-Coumaroyl) harpagide

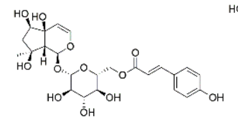

6'-O-(*p*-Coumaroyl) harpagide

88

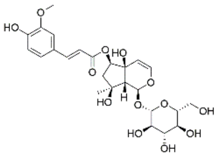

94 6-O-*trans*-Feruloylharpagide

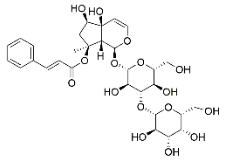

98 Ningposide II

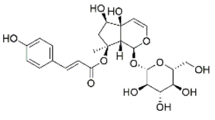

102 8-O-*p*-Coumaroylharpagide

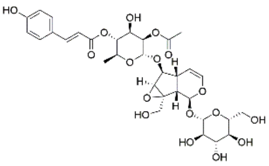

103 Scrophuloside A2

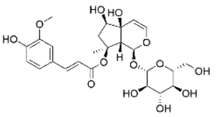

105 8-O-*trans*-Feruloylharpagide

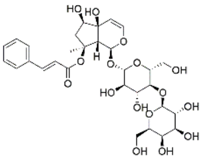

107 Ningposide I

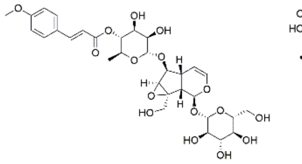

108 Verbascoside A

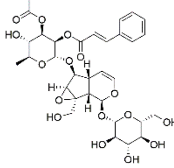

109 Scorodioside

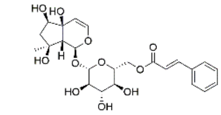

114 6'-O-cinnamoylharpagide

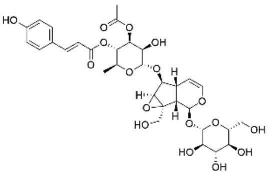

115 Scrophuloside A3

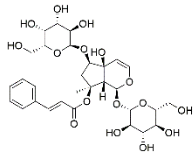

117 6-O-*α*-*D*-galactopyranosylharpagoside

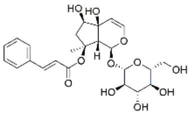

118 Harpagoside

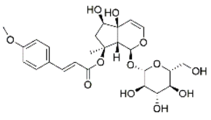

119 8-O-methoxycinnamoylharpagide

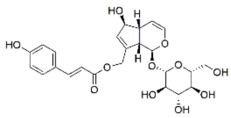

120 Eurostoside

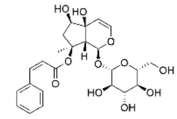

121 *Z*-Harpagoside

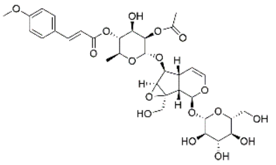

122 Scrophuloside A5

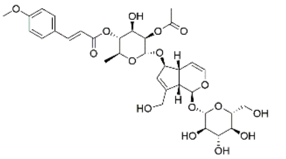

123 Buddlejoside A2

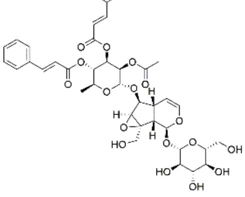

128 Scropolioside B

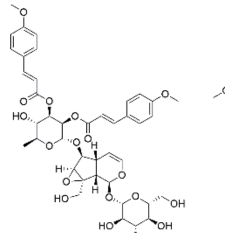

130 Scrophuloside A9

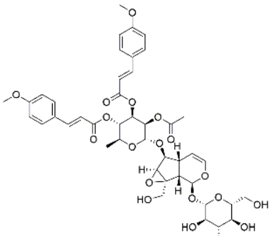

131 Scropolioside A4

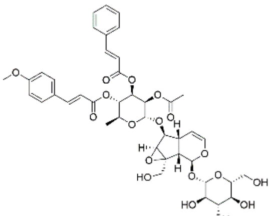

132 Scrophuloside B4

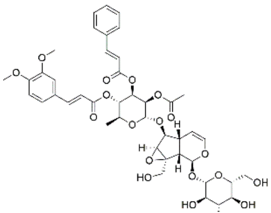

133 Methoxylscrophuloside B4

Figure S4. The 52 identified iridoid glycosides in *S. ningpoensis* (continued).

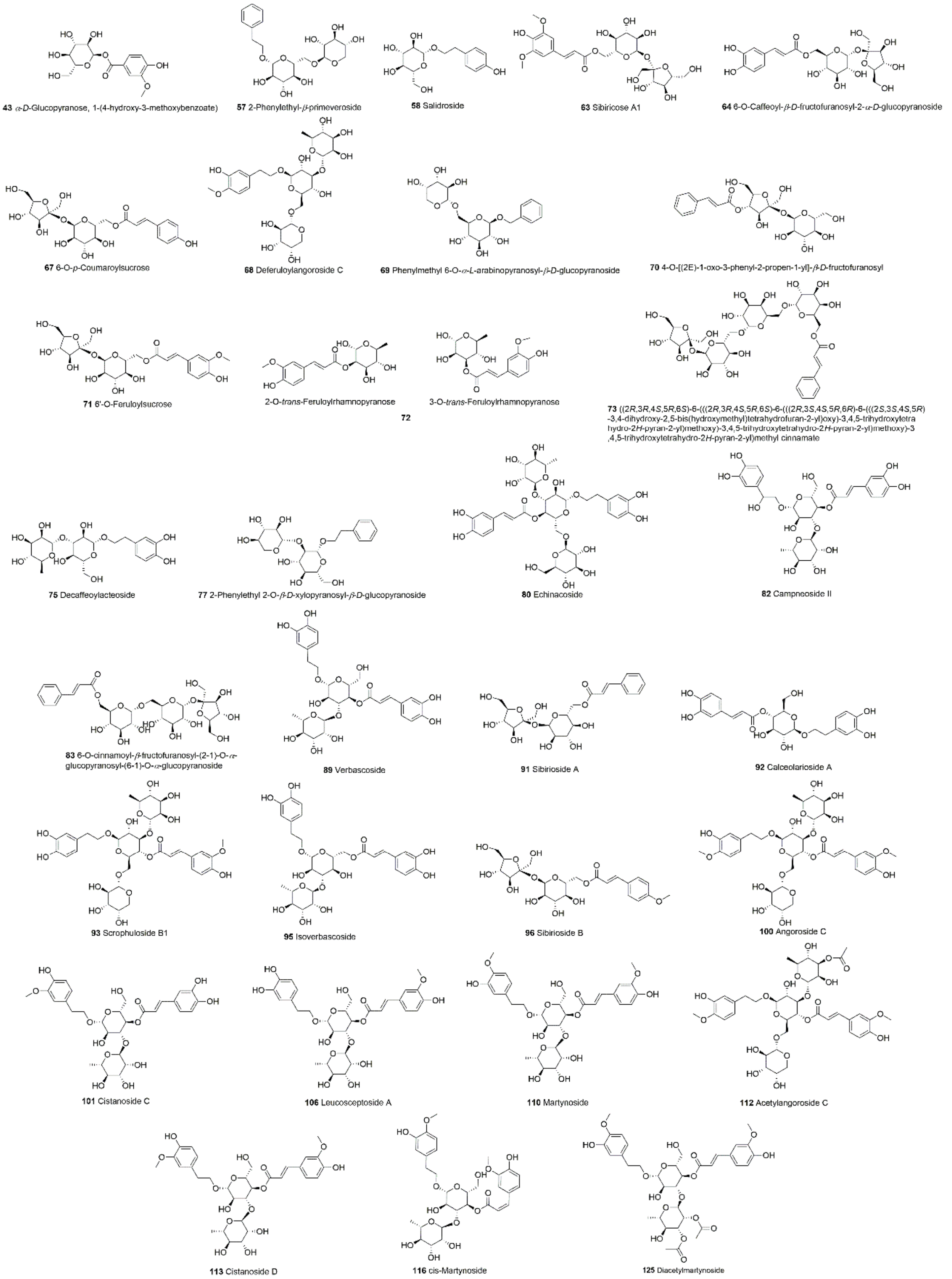

Figure S5. The 31 identified phenylpropanoid glycosides in *S. ningpoensis*.

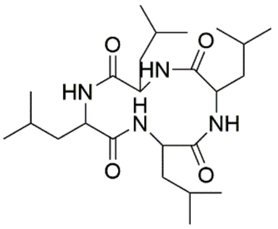

78 Cyclotetraleucyl (isoleucyl)

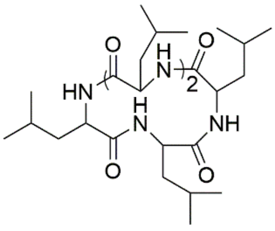

86 Cyclopentaleucyl (isoleucyl)

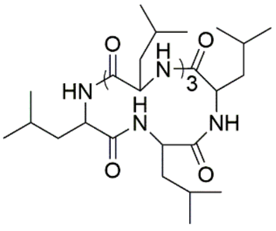

97 Cyclohexaleucyl(isoleucyl)

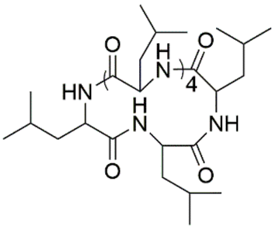

99 Cycloheptaleucyl (isoleucyl)

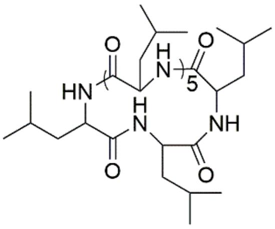

140 Cyclooctaleucyl (isoleucyl)

Figure S6. The 5 identified cyclopeptides in *S. ningpoensis.*

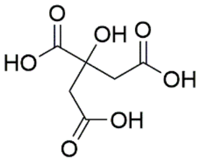

19 Citric acid

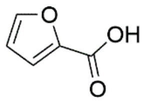

20 Furan-2-carboxylic acid

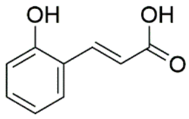

81 2-Hydroxycinnamic acid

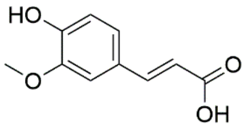

85 Ferulic acid

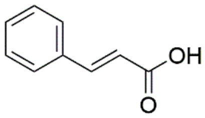

124 Cinnamic acid

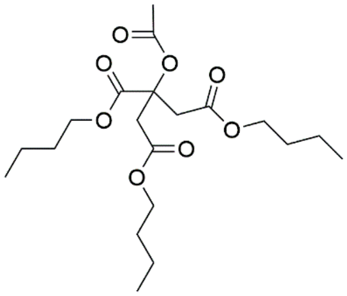

135 Acetyl tributyl citrate

Figure S7. The 6 identified organic acids in *S. ningpoensis.*

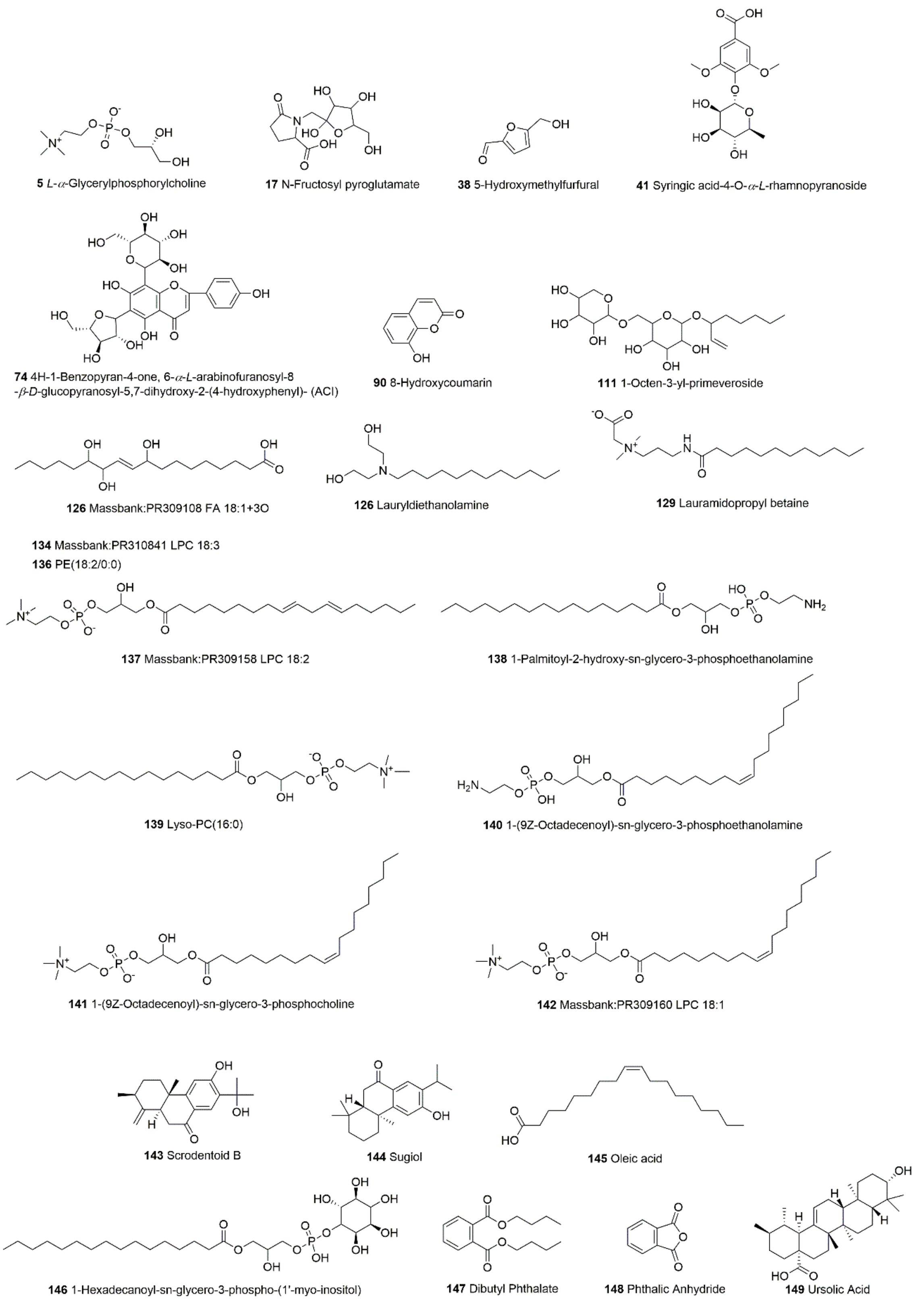

Figure S8. The 25 identified other compounds in *S. ningpoensis*.

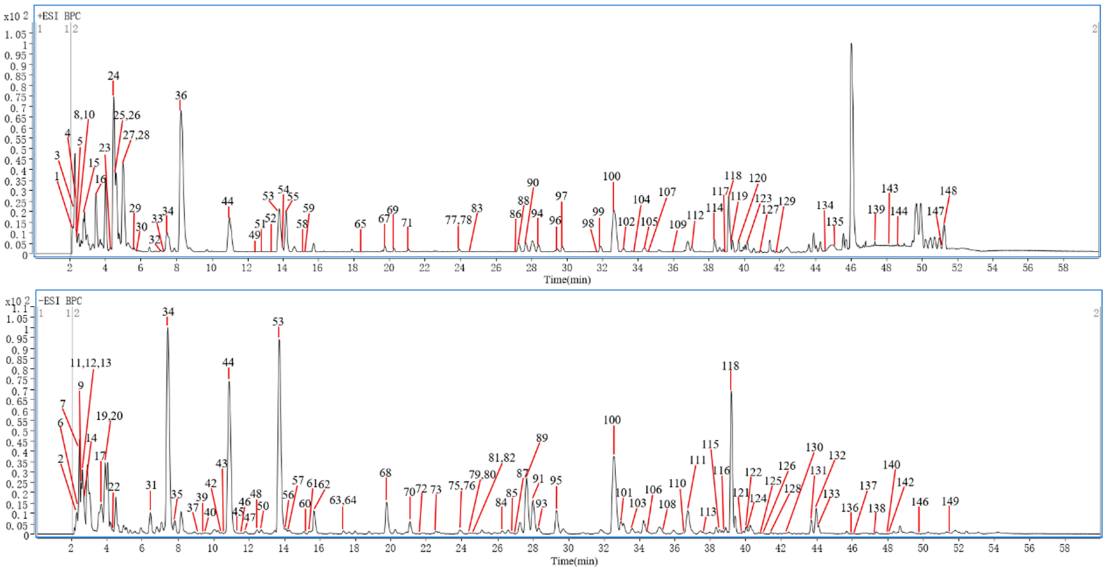

Figure S9. The BPC of S2 by UHPLC-Q-TOF-MS in both positive ion mode and negative ion mode.

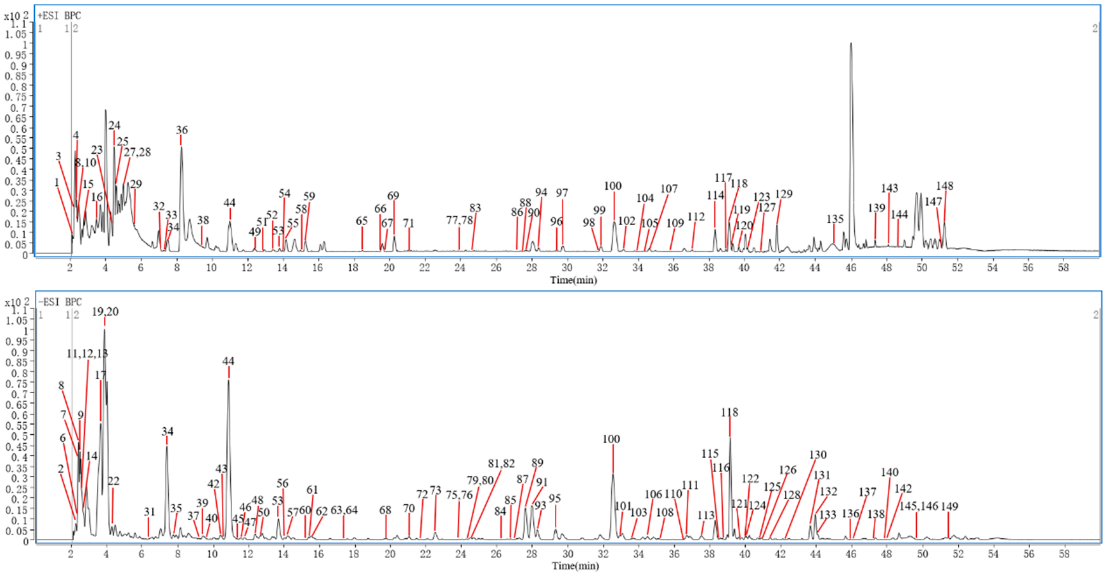

Figure S10. The BPC of S3 by UHPLC-Q-TOF-MS in both positive ion mode and negative ion mode.

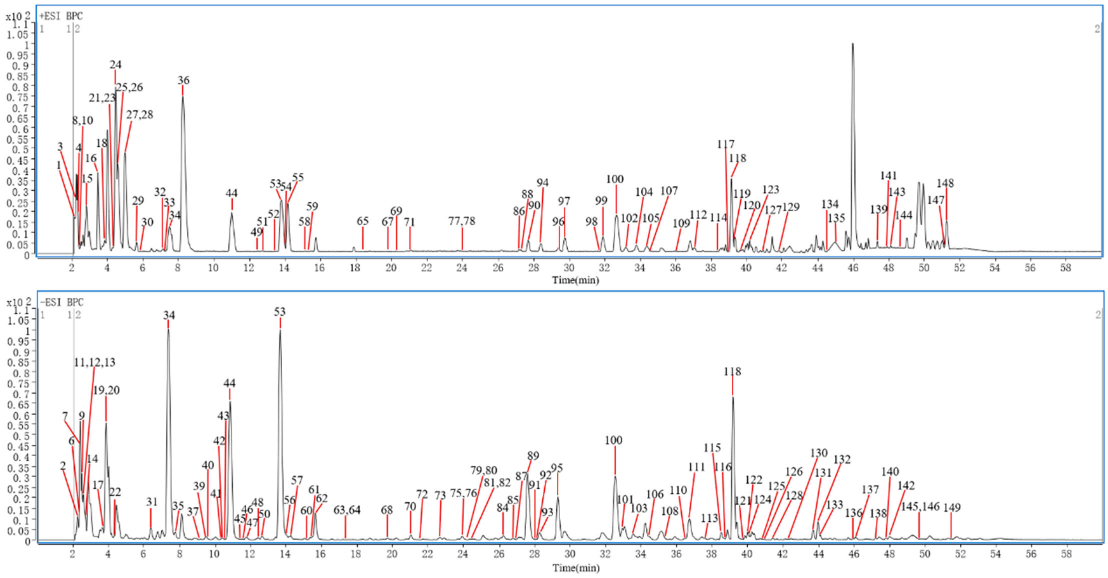

Figure S11. The BPC of S4 by UHPLC-Q-TOF-MS in both positive ion mode and negative ion mode.

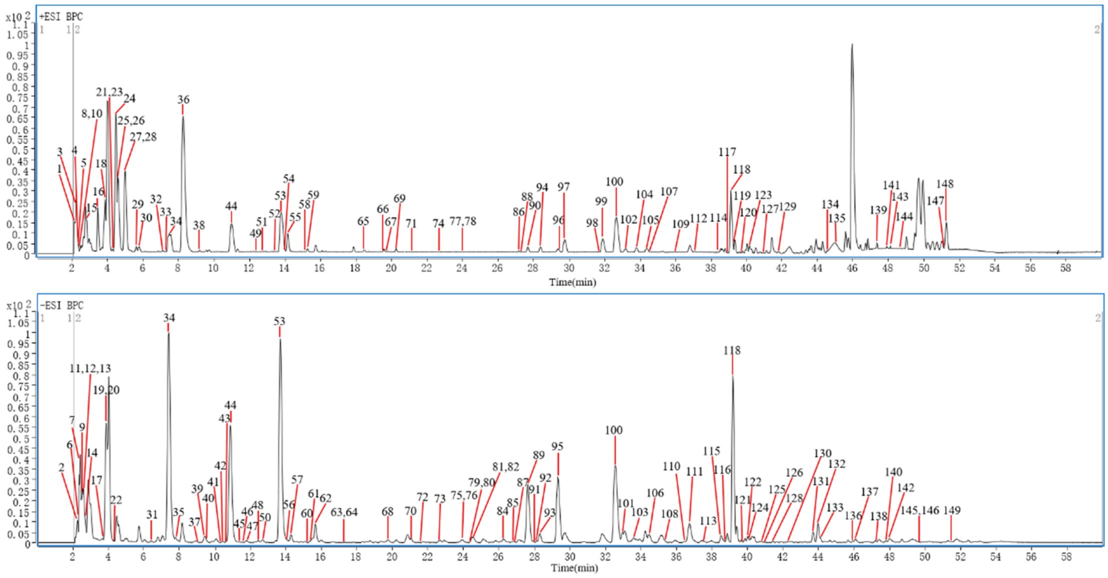

Figure S12. The BPC of S5 by UHPLC-Q-TOF-MS in both positive ion mode and negative ion mode.

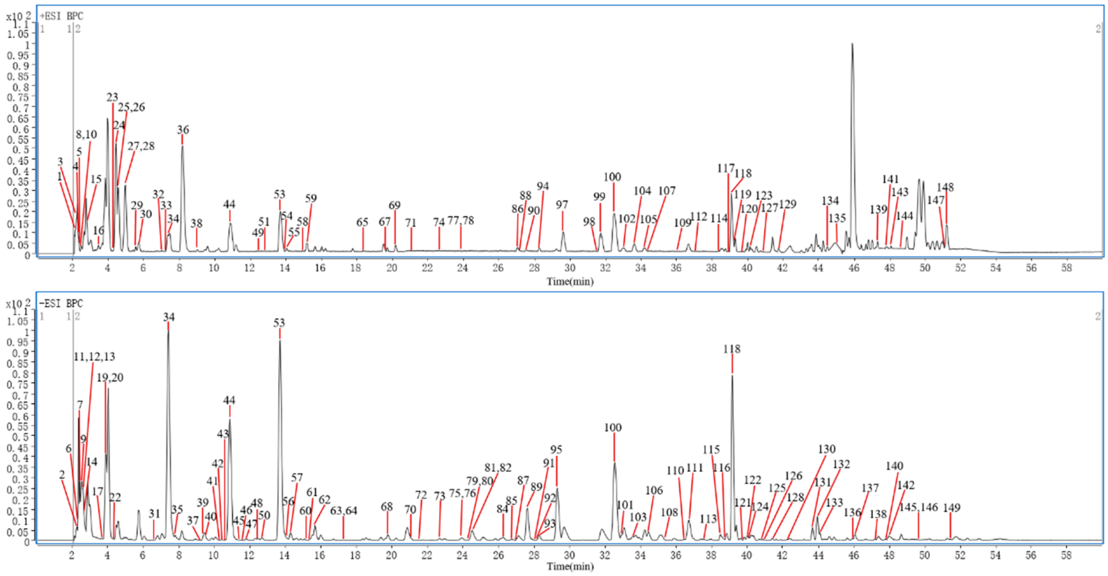

Figure S13. The BPC of S6 by UHPLC-Q-TOF-MS in both positive ion mode and negative ion mode.

Table S1. Classification and Identification of the metabolites of SR by UHPLC-Q-TOF-MS.

No.	R.T. (min)	Identification	Formula	Error （ppm）	Adducts	MS	MS^2 ions	Classification	S1	S13	S24	S62	S66	S67
**1**	2.147	Trimethyllysine	$C_9H_{20}N_2O_2$	4.47	$[M+H]^+$	189.1606	175.1196,147.1172, 130.0875	Amino acid	✓	✓	✓	✓	✓	✓
**2**	2.217	Histidine	$C_6H_9N_3O_2$	1.95	$[M-H]^-$	154.0625	110.1726	Amino acid	✓	✓	✓	✓	✓	✓
**3**	2.219	Arginine	$C_6H_{14}N_4O_2$	3.7	$[M+H]^+$	175.1196	116.0706	Amino acid	✓	✓	✓	✓	✓	✓
**4**	2.315	Glutamine	$C_5H_{10}N_2O_3$	2.59	$[M+H]^+$	147.0768	135.0503,120.0658	Amino acid	✓	✓	✓	✓	✓	✓
**5**	2.317	*L*-*α*-Glycerylphosphoryl choline	$C_8H_{20}NO_6P$	3.49	$[M+H]^+$	258.111	184.0738,104.0709	Others	✓	✓			✓	✓
**6**	2.391	Isomaltulose	$C_{12}H_{22}O_{11}$	3.12	$[M-H]^-$	341.11	323.0997,179.0564	Saccharide	✓	✓	✓	✓	✓	✓
**7**	2.434	*L*-Arabinose	$C_5H_{10}O_5$	-2.94	$[M+HCOO]^-$	195.0516	165.0406	Saccharide	✓	✓	✓	✓	✓	✓
**8**	2.447	*D*-1-[(3-Carboxypropyl) amino]-1-deoxyfructose	$C_{10}H_{19}NO_7$	3.65	$[M+H]^+$	266.1244	248.1135,116.0709	Amino acid	✓	✓	✓	✓	✓	✓
**9**	2.472	Stachyose	$C_{24}H_{42}O_{21}$	-0.12	$[M-H]^-$	665.2145	503.1622,341.1090, 161.0455	Saccharide	✓	✓	✓	✓	✓	✓

**10**	2.531	2'-O-Methyladenosine	$C_{11}H_{15}N_5O_4$	1.7	$[M+H]^+$	282.12	264.1083	Nucleoside	✓	✓	✓	✓	✓	✓
**11**	2.578	Sucrose	$C_{12}H_{22}O_{11}$	0.74	$[M+HCOO]^-$	387.1147	179.0563	Saccharide	✓	✓	✓	✓	✓	✓
**12**	2.602	Raffinose	$C_{18}H_{32}O_{16}$	1.21	$[M+HCOO]^-$	549.1679	387.1150,341.1097	Saccharide	✓	✓	✓	✓	✓	✓
**13**	2.626	Trehalose	$C_{12}H_{22}O_{11}$	1	$[M+HCOO]^-$	387.1148	341.1093,179.0562	Saccharide	✓	✓	✓	✓	✓	✓
**14**	2.722	Fructose/Glucose	$C_6H_{12}O_6$	2.17	$[M-H]^-$	179.0565	161.0452	Saccharide	✓	✓	✓	✓	✓	✓
**15**	2.975	Valine	$C_5H_{11}NO_2$	0.38	$[M+H]^+$	118.0863	72.0809	Amino acid	✓	✓	✓	✓	✓	✓
**16**	3.491	Methionine	$C_5H_{11}NO_2S$	2.49	$[M+H]^+$	150.0587	87.0262	Amino acid	✓	✓	✓	✓	✓	✓
**17**	3.646	N-Fructosyl pyroglutamate	$C_{11}H_{17}NO_8$	2.96	$[M-H]^-$	290.089	200.0570,128.0353	Others	✓	✓	✓	✓	✓	✓
**18**	3.776	Adenosine 2',3'-cyclic monophosphate	$C_{10}H_{12}N_5O_6P$	2.13	$[M+H]^+$	330.0605	136.0618	Nucleoside				✓	✓	
**19**	3.886	Citric acid	$C_6H_8O_7$	2.48	$[M-H]^-$	191.0202	173.0092,147.0300, 120.0195	Organic acid	✓	✓	✓	✓	✓	✓
**20**	3.889	Furan-2-carboxylic acid	$C_5H_4O_3$	0.29	$[M-H]^-$	111.0088	67.0188	Organic acid	✓	✓	✓	✓	✓	✓

**21**	4.304	Guanosine cyclic monophosphate	$C_{10}H_{12}O_7N_5P$	2.28	$[M+H]^+$	346.0555	152.0567,135.0800	Nucleoside	✓			✓	✓	
**22**	4.307	Arabinofuranosyluracil	$C_9H_{12}N_2O_6$	2.63	$[M-H]^-$	243.0629	219.0885,111.0082,	Nucleoside	✓	✓	✓	✓	✓	✓
**23**	4.334	5'-Deoxy-5'-(methylsulfinyl)adenosine	$C_{11}H_{15}N_5O_4S$	-1.75	$[M+H]^+$	314.0923	136.0753	Nucleoside	✓	✓	✓	✓	✓	✓
**24****	4.454	Tyrosine	$C_9H_{11}NO_3$	4.56	$[M+H]^+$	182.082	136.0755	Amino acid	✓	✓	✓	✓	✓	✓
**25**	4.584	Isoleucine	$C_6H_{13}NO_2$	1.48	$[M+H]^+$	132.1021	86.0964	Amino acid	✓	✓	✓	✓	✓	✓
**26**	4.593	Catalpol	$C_{15}H_{22}O_{10}$	1.25	$[M+Na]^+$	385.111	344.1342,182.0813	Iridoid	✓	✓		✓	✓	✓
**27**	4.92	Leucine	$C_6H_{13}NO_2$	1.48	$[M+H]^+$	132.1021	96.0962	Amino acid	✓	✓	✓	✓	✓	✓
**28**	4.947	Adenosine	$C_{10}H_{13}N_5O_4$	2.87	$[M+H]^+$	268.1046	136.0619,119.0352	Nucleoside	✓	✓	✓	✓	✓	✓
**29**	5.629	Guanosine	$C_{10}H_{13}N_5O_5$	3.36	$[M+H]^+$	284.0999	184.9852,167.0694, 158.9612,140.9615	Nucleoside	✓	✓	✓	✓	✓	✓
**30**	5.799	7,8-Dehydroharpagide	$C_{15}H_{22}O_9$	1.35	$[M+NH_4]^+$	364.1607	167.0698,149.0598	Iridoid	✓	✓	✓	✓	✓	✓
**31**	6.384	Ajugol	$C_{15}H_{24}O_9$	1.44	$[M+HCOO]^-$	393.1408	185.0817,167.0700	Iridoid	✓	✓	✓	✓	✓	✓
**32**	7.082	6'-O-*β*-Glucosylaucubin	$C_{21}H_{32}O_{14}$	0.89	$[M+Na]^+$	531.1689	369.1155,351.1048, 203.0529, 131.0486	Iridoid	✓	✓	✓	✓	✓	✓

**33**	7.31	6-O-*β*-Glucosylaucubin	$C_{21}H_{32}O_{14}$	2.02	$[M+Na]^+$	531.1695	369.1163,130.9665	Iridoid	✓	✓	✓	✓	✓	✓
**34****	7.541	Aucubin	$C_{15}H_{22}O_9$	-0.82	$[M+Na]^+$	369.1153	207.0622	Iridoid	✓	✓	✓	✓	✓	✓
**35**	7.91	Dihydroharpagide	$C_{15}H_{26}O_{10}$	1.46	$[M+HCOO]^-$	411.1514	311.1358,203.0844, 160.8431	Iridoid	✓	✓	✓	✓	✓	
**36****	8.259	Phenylalanine	$C_9H_{11}NO_2$	2.08	$[M+H]^+$	166.0866	120.0811	Amino acid	✓	✓	✓	✓	✓	✓
**37**	9.073	Monomelittoside	$C_{15}H_{22}O_{10}$	-0.98	$[M+HCOO]^-$	407.1191	199.0605,160.8427	Iridoid	✓	✓	✓	✓	✓	✓
**38**	9.221	5-Hydroxymethylfurfural	$C_6H_6O_3$	0.23	$[M+H]^+$	127.039	81.0328	Others			✓		✓	✓
**39**	9.423	Ningpogoside A/Ningpogoside B	$C_{15}H_{24}O_8$	2.33	$[M+HCOO]^-$	377.1462	168.888	Iridoid	✓	✓	✓	✓	✓	✓
**40**	9.53	*β-D*-Glucopyranoside,1,4a,5,7a-tetrahydro-5-hydroxy-7-(hydroxymethyl)cyclopenta[c]pyran-1-yl 6-O-*α-D*-xylopyranosyl-, [1S-	$C_{20}H_{30}O_{13}$	1.06	$[M+HCOO]^-$	523.1674	345.1188,183.0672, 165.0560,161.0419, 131.0349	Iridoid	✓	✓	✓	✓	✓	✓

		(1α,4aα,5α,7aα)] -(9CI)												
**41**	10.268	Syringic acid-4-O-α-*L*-rhamnopyranoside	$C_{15}H_{20}O_9$	-0.35	$[M+HCOO]^-$	389.1088	315.0730,145.0507	Others				✓	✓	✓
**42**	10.383	6-O-β-Glucosylaucubin isomer	$C_{21}H_{32}O_{14}$	1.32	$[M-H]^-$	507.1726	391.1246,229.8743,183.0292,160.8414	Iridoid	✓	✓	✓	✓	✓	✓
**43**	10.575	*α-D*-Glucopyranose,1-(4-hydroxy-3-methoxybenzoate)	$C_{14}H_{18}O_9$	0.29	$[M-H]^-$	329.0879	167.0342,160.8434	Phenylpropanoid	✓	✓	✓	✓	✓	✓
**44**[**]	10.976	Harpagide	$C_{15}H_{24}O_{10}$	1.37	$[M+Na]^+$	387.1267	369.1158,225.0736,207.0629,189.0523	Iridoid	✓	✓	✓	✓	✓	✓
**45**	11.392	Dihydrocatalpol	$C_{15}H_{24}O_{10}$	1.83	$[M+HCOO]^-$	409.1359	201.0766,183.0695	Iridoid	✓	✓	✓	✓	✓	✓
**46**	11.56	6-O-Glucosylharpagide isomer	$C_{21}H_{34}O_{15}$	1.1	$[M+HCOO]^-$	571.1886	363.1323,201.0786,183.0671,160.8436	Iridoid	✓	✓	✓	✓	✓	✓
**47**[*]	11.824	*α-L*-Rhamnopyranoside, (1*S*,4a*S*,5*R*,7*S*,7a*R*)-1,4a,5,6,7,7a-	$C_{15}H_{24}O_9$	2.45	$[M+HCOO]^-$	393.1412	201.0766,183.0659,165.0561,162.8395	Iridoid	✓	✓	✓	✓	✓	✓

		hexahydro-4a,5,7-trihydroxy-7-methylcyclopenta[c] pyran-1-yl (9CI, ACI)												
**48***	12.416	(2*R*,3*S*,4*S*,5*R*,6*R*)-2-(Hydroxymethyl)-6-(((2*R*,3*S*,4*S*,5*R*,6*S*)-3,4,5-trihydroxy-6-(((1a*S*,1b*S*,2*S*,5a*R*,6*S*,6a*S*)-1a-(hydroxymethyl)-6-methoxy-1a,1b,2,5a,6,6a-hexahydrooxireno [2',3':4,5] cyclopenta [1,2-c] pyran-2-yl) oxy) tetrahydro-2H-pyran-2-yl) methoxy) tetrahydro-2H-pyran-3,4,5-triol	$C_{22}H_{34}O_{15}$	0.9	$[M+HCOO]^-$	583.1885	421.0402,323.0981, 213.07062,195.0664 165.0592,161.0457	Iridoid	✓	✓	✓	✓	✓	✓
**49**	12.485	6'-O-Glucosylharpagide	$C_{21}H_{34}O_{15}$	0.74	$[M+Na]^+$	549.1794	531.1775,365.1053, 167.0702	Iridoid	✓	✓	✓	✓	✓	✓

**50***	12.7	(2*R*,3*S*,4*S*,5*R*,6*R*)-2-(Hydroxymethyl)-6-(((2*R*,3*S*,4*S*,5*R*,6*S*)-3,4,5-trihydroxy-6-(((1a*S*,1b*S*,2*S*,5a*R*,6*S*,6a*S*)-1a-(hydroxymethyl)-6-methoxy-1a,1b,2,5a,6,6a-hexahydrooxireno[2',3':4,5]cyclopenta[1,2-c]pyran-2-yl) oxy) tetrahydro-2H-pyran-2-yl) methoxy) tetrahydro-2H-pyran-3,4,5-triol isomer	$C_{22}H_{34}O_{15}$	0.9	$[M+HCOO]^-$	583.1885	421.0402,323.0981, 213.07062,195.0664 165.0592,161.0457	Iridoid	✓	✓	✓	✓	✓	✓
**51**	12.773	6-O-Glucosylharpagide	$C_{21}H_{34}O_{15}$	-0.35	$[M+Na]^+$	549.1788	5531.1699,387.1262 ,369.1158,369.1051	Iridoid	✓	✓	✓	✓	✓	✓
**52**	13.434	1-*β*-*D*-Glucopyranosyl-*L*-tryptophan	$C_{17}H_{22}O_7N_2$	1.97	$[M+H]^+$	367.1507	143.9595,130.0083, 118.0862	Amino acid		✓	✓	✓	✓	

**53****	13.786	6-O-Methyl-catalpol	$C_{16}H_{24}O_{10}$	0.58	$[M+Na]^+$	399.1264	237.0736,219.0449,187.0183	Iridoid	✓	✓	✓	✓	✓	✓
**54**	14.011	Methylthioadenosine	$C_{11}H_{15}N_5O_3S$	-2.23	$[M+H]^+$	298.0975	136.0620,119.0496	Nucleoside	✓	✓	✓	✓	✓	✓
**55****	14.191	Tryptophan	$C_{11}H_{12}N_2O_2$	2.17	$[M+H]^+$	205.0976	188.0706,118.0861	Amino acid	✓	✓	✓	✓	✓	✓
**56**	14.24	3,4-Dihydromethylcatalpol	$C_{16}H_{26}O_{10}$	0.71	$[M+HCOO]^-$	423.1511	215.0924,197.0835,179.0715	Iridoid	✓	✓	✓	✓	✓	✓
**57**	14.351	2-Phenylethyl-*β*-primeveroside	$C_{19}H_{28}O_{10}$	1.19	$[M+HCOO]^-$	461.167	178.9776,165.0913	Phenylpropanoid	✓	✓	✓	✓	✓	✓
**58**	15.116	Salidroside	$C_{14}H_{20}O_7$	2.74	$[M+NH_4]^+$	318.1556	283.0811,121.0638	Phenylpropanoid	✓	✓	✓	✓	✓	✓
**59**	15.260/16.245	Ningpoensine B/Ningpoensine C	$C_{15}H_{21}NO_4$	-4.87	$[M+H]^+$	280.1557	262.2387,222.1125	Iridoid	✓	✓	✓	✓	✓	✓
**60**	15.162	6-O-*α*-*L*-Rhamnopyranosylcatalpol	$C_{21}H_{32}O_{14}$	-0.02	$[M+HCOO]^-$	553.1774	345.1200,162.8391,160.8427,145.0509	Iridoid	✓	✓	✓	✓	✓	✓
**61**	15.39	6-O-*α*-*L*-Rhamnopyranosylaucubin	$C_{21}H_{32}O_{13}$	0.57	$[M+HCOO]^-$	537.1828	537.1828,183.0660,161.0449	Iridoid	✓	✓	✓	✓	✓	✓

**62**	15.63	6-O-Methyl-aucubin	$C_{16}H_{24}O_{9}$	1.64	$[M+HCOO]^{-}$	405.1409	197.0809,161.0444	Iridoid	✓	✓	✓	✓	✓	✓
**63**	17.299	Sibiricose A1	$C_{23}H_{32}O_{15}$	1.2	$[M-H]^{-}$	547.1675	385.1113,341.0887, 160.8421	Phenylpropanoid	✓	✓	✓	✓	✓	✓
**64**	17.432	6-O-Caffeoyl-*β*-*D*-fructofuranosyl-2-*α*-*D*-glucopyranoside	$C_{21}H_{28}O_{14}$	1.93	$[M-H]^{-}$	503.1416	341.0891,178.9765	Phenylpropanoid	✓	✓	✓	✓	✓	✓
**65**	18.468	5-Methoxy-*L*-tryptophan	$C_{12}H_{14}N_{2}O_{3}$	1.59	$[M-H2O+H]^{+}$	217.0975	144.0805,127.0539	Amino acid	✓	✓	✓	✓	✓	✓
**66**	19.441	1-Methyl-1,2,3,4-tetrahydro-*β*-carboline-3-carboxylic acid	$C_{13}H_{14}N_{2}O_{2}$	2.58	$[M+H]^{+}$	231.1134	158.0963	Amino acid			✓		✓	
**67**	19.718	6-O-p-Coumaroylsucrose	$C_{21}H_{28}O_{13}$	-1.59	$[M+Na]^{+}$	511.1414	349.0898,147.0440	Phenylpropanoid	✓	✓	✓	✓	✓	✓
**68**	19.725	Deferuloylangoroside C	$C_{26}H_{40}O_{16}$	0.73	$[M-H]^{-}$	607.2248	561.1440,475.1826, 160.8424	Phenylpropanoid	✓	✓	✓	✓	✓	✓
**69**	20.207	Phenylmethyl 6-O-*α*-*L*-arabinopyranosyl-*β*-*D*-glucopyranoside	$C_{18}H_{26}O_{10}$	0.9	$[M+NH_4]^{+}$	420.1868	239.1502,204.9849, 185.0783,163.0961	Phenylpropanoid	✓	✓	✓	✓	✓	✓

**70**	21.033	4-O-[(2*E*)-1-oxo-3-Phenyl-2-propen-1-yl]-*β*-*D*-fructofuranosyl	$C_{21}H_{28}O_{12}$	-1.89	$[M+HCOO]^-$	517.1553	355.1080,337.0925,193.0508,175.0403	Phenylpropanoid	✓	✓	✓	✓	✓	✓
**71**	21.072	6'-O-Feruloylsucrose	$C_{22}H_{30}O_{14}$	2.26	$[M+Na]^+$	541.154	357.1184,217.1055,177.0548,163.0959,145.0287	Phenylpropanoid	✓	✓	✓	✓	✓	✓
**72**	21.643	2-O-*trans*-Feruloylrhamnopyranose/3-O-*trans*-Feruloylrhamnopyranose	$C_{16}H_{20}O_8$	2.53	$[M-H]^-$	339.1094	321.2070,145.0498	Phenylpropanoid	✓	✓	✓	✓	✓	✓
**73**[*]	22.519	((2*R*,3*R*,4*S*,5*R*,6*S*)-6-(((2*R*,3*R*,4*S*,5*R*,6*S*)-6-(((2*R*,3*S*,4*S*,5*R*,6*R*)-6-(((2*S*,3*S*,4*S*,5*R*)-3,4-Dihydroxy-2,5-bis(hydroxymethyl)tetrahydrofuran-2-yl)oxy)-3,4,5-trihydroxytetrahydro-2H-pyran-2-	$C_{33}H_{48}O_{22}$	0.19	$[M-H]^-$	795.2566	647.2039, 341.1091,323.0994,179.0556,147.0452,103.0557	Phenylpropanoid	✓	✓	✓	✓	✓	✓

		yl)methoxy)-3,4,5-trihydroxytetrahydro-2H-pyran-2-yl)methoxy)-3,4,5-trihydroxytetrahydro-2H-pyran-2-yl)methyl cinnamate												
**74**	22.778	4H-1-Benzopyran-4-one, 6-*α*-*L*-arabinofuranosyl-8-*β*-*D*-glucopyranosyl-5,7-dihydroxy-2-(4-hydroxyphenyl)-(ACI)	$C_{26}H_{28}O_{14}$	0.92	$[M+H]^+$	565.1557	325.0928,179.9913, 150.0471	Others					✓	✓
**75**	23.771	Decaffeoylacteoside	$C_{20}H_{30}O_{12}$	0.33	$[M-H]^-$	461.1666	263.1242,161.0498, 135.0559,125.0266	Phenylpropanoid	✓	✓	✓	✓	✓	✓
**76**	23.916	Saccatoside	$C_{30}H_{38}O_{16}$	-1.08	$[M-H]^-$	653.208	491.1560,473.1431, 181.0506,163.0400, 145.0294	Iridoid	✓	✓	✓	✓	✓	✓
**77**	23.918	2-Phenylethyl 2-O-*β*-*D*-xylopyranosyl-*β*-*D*-glucopyranoside	$C_{19}H_{28}O_{10}$	1.68	$[M+NH_4]^+$	434.2028	145.0495,133.0508, 127.0388,115.0390, 97.0286	Phenylpropanoid	✓	✓	✓	✓	✓	✓

**78**	23.932	Cyclotetraleucyl (isoleucyl)	$C_{24}H_{44}N_4O_4$	1.91	$[M+H]^+$	453.3444	435.2058	Cyclopeptide	✓	✓	✓	✓	✓	✓
**79**	24.288	6-O-Caffeoylharpagide/6'-O-Caffeoylharpagide	$C_{24}H_{30}O_{13}$	2.92	$[M-H]^-$	525.1629	178.9776,160.8431	Iridoid	✓	✓	✓	✓	✓	✓
**80**	24.399	Echinacoside	$C_{35}H_{46}O_{20}$	0.55	$[M-H]^-$	785.2514	623.1959,461.1655, 179.0364,161.0217	Phenylpropanoid	✓	✓	✓	✓	✓	✓
**81**	24.528	2-Hydroxycinnamic acid	$C_9H_8O_3$	2.65	$[M-H]^-$	163.0405	119.0504	Organic acid	✓	✓	✓	✓	✓	✓
**82**	24.62	Campneoside II	$C_{29}H_{36}O_{16}$	2.1	$[M-H]^-$	639.1944	621.1829,459.1508, 179.0353,161.0245	Phenylpropanoid	✓	✓	✓	✓	✓	✓
**83**	24.725	6-O-Cinnamoyl-*β*-fructofuranosyl-(2-1)-O-*α*-glucopyranosyl-(6-1)-O-*α*-glucopyranoside	$C_{27}H_{38}O_{17}$	-0.03	$[M+Na]^+$	657.2001	527.0824,455.1566, 185.0782,149.0448, 130.9661	Phenylpropanoid		✓	✓			
**84**	26.245	6-O-*α*-*L*-(2″-O-feruloyl) rhamnopyranosyl-catalpol	$C_{31}H_{40}O_{17}$	-1.72	$[M-H]^-$	683.2181	407.1358,193.0506, 175.0400,145.0273	Iridoid	✓	✓	✓	✓	✓	✓

**85**	26.783	Ferulic acid	$C_{10}H_{10}O_4$	2.94	$[M-H]^-$	193.0512	174.9560,149.0600	Organic acid	✓	✓	✓	✓	✓	✓
**86**	27.017	Cyclopentaleucyl (isoleucyl)	$C_{30}H_{55}N_5O_5$	0.54	$[M+H]^+$	566.4279	548.4175,435.3340, 322.2493,209.1650, 96.0807	Cyclopeptide	✓	✓	✓	✓	✓	✓
**87**	27.163	1*β*-Hydroxy-6*β*-methoxy-dihydrocatalpolgenin/1*α*-Hydroxy-6*β*-methoxy-dihydrocatalpolgenin	$C_{10}H_{16}O_5$	0.09	$[M+HCOO]^-$	261.098	178.9779	Iridoid	✓	✓	✓	✓	✓	✓
**88**	27.293	6-O-(p-Coumaroyl) harpagide/6'-O-(p-coumaroyl) harpagide	$C_{24}H_{30}O_{12}$	1.41	$[M+Na]^+$	533.1637	186.9570,167.0707	Iridoid	✓	✓	✓	✓	✓	✓
**89****	27.59	Verbascoside	$C_{29}H_{36}O_{15}$	0.89	$[M-H]^-$	623.1987	461.1670,315.1093, 179.0356,161.0249	Phenylpropanoid	✓	✓	✓	✓	✓	✓
**90**	27.668	8-Hydroxycoumarin	$C_9H_6O_3$	0.79	$[M+H]^+$	163.0391	135.0438,117.0331, 89.0384	Others	✓	✓	✓	✓	✓	✓
**91**	27.995	Sibirioside A	$C_{21}H_{28}O_{12}$	0.81	$[M+HCOO]^-$	517.1567	323.0985,161.0606, 147.0453	Phenylpropanoid	✓	✓	✓	✓	✓	✓
**92**	28.111	Calceolarioside A	$C_{23}H_{26}O_{11}$	-1.12	$[M-H]^-$	477.1397	178.9774,160.8432	Phenylpropanoid				✓	✓	✓

**93**	28.251	Scrophuloside B1	$C_{35}H_{46}O_{19}$	0.84	$[M-H]^-$	769.2567	637.2139,575.1533	Phenylpropanoid	✓	✓	✓	✓	✓	✓
**94**	28.385	6-O-*trans*-Feruloylharpagide	$C_{25}H_{32}O_{13}$	1.22	$[M+Na]^+$	563.1742	379.0999,177.0543	Iridoid	✓	✓	✓	✓	✓	✓
**95**[**]	29.295	Isoverbascoside	$C_{29}H_{36}O_{15}$	0.25	$[M-H]^-$	623.1997	461.1664,315.1083, 179.0354,161.0246	Phenylpropanoid	✓	✓	✓	✓	✓	✓
**96**	29.442	Sibirioside B	$C_{22}H_{30}O_{13}$	-0.88	$[M+Na]^+$	525.1574	365.1110,363.1052, 203.0524,185.0420, 161.0599	Phenylpropanoid	✓	✓	✓	✓	✓	
**97**	29.512	Cyclohexaleucyl (isoleucyl)	$C_{36}H_{66}N_6O_6$	0.35	$[M+H]^+$	679.5119	661.5011,548.4172, 435.3336,322.2490, 209.1650,96.0807	Cyclopeptide	✓	✓	✓	✓	✓	✓
**98**	31.592	Ningposide II	$C_{30}H_{40}O_{16}$	-0.08	$[M+Na]^+$	679.2208	661.2108,185.0790, 149.0597,130.9661	Iridoid	✓	✓	✓	✓	✓	✓
**99**	31.729	Cycloheptaleucyl (isoleucyl)	$C_{42}H_{77}N_7O_7$	0.6	$[M+H]^+$	792.5962	774.5851,661.5011, 548.4180,435.3335, 322.2490,209.1649, 96.0806	Cyclopeptide	✓	✓	✓	✓	✓	✓
**100**[**]	32.612	Angoroside C	$C_{36}H_{48}O_{19}$	0.63	$[M-H]^-$	783.2722	607.2245,589.2137, 443.1566,193.0509, 175.0405	Phenylpropanoid	✓	✓	✓	✓	✓	✓
**101**	32.898	Cistanoside C	$C_{30}H_{38}O_{15}$	0.32	$[M-H]^-$	637.214	475.1819,329.1238, 179.0353,161.0247	Phenylpropanoid	✓	✓	✓	✓	✓	✓

**102**	33.164	8-O-p-Coumaroylharpagide	$C_{24}H_{30}O_{12}$	-1.78	$[M+Na]^+$	533.162	369.1158,351.1052, 203.0523,189.0519	Iridoid	✓	✓	✓	✓	✓	✓
**103**	33.498	Scrophuloside A2	$C_{32}H_{40}O_{17}$	0.04	$[M-H]^-$	695.2193	533.1657,515.1548, 181.0498,163.0399, 145.0296	Iridoid	✓	✓	✓	✓	✓	✓
**104**	33.602	Cyclooctaleucyl (isoleucyl)	$C_{48}H_{88}N_8O_8$	-0.54	$[M+H]^+$	905.6793	887.6677,774.5860, 661.5018,548.4165, 435.3340,322.2488, 209.1645	Cyclopeptide	✓	✓	✓	✓	✓	✓
**105**	34.341	8-O-*trans*-Feruloylharpagide	$C_{25}H_{32}O_{13}$	1.22	$[M+Na]^+$	563.1742	225.0702,177.0543	Iridoid	✓	✓	✓	✓	✓	✓
**106**	34.484	Leucosceptoside A	$C_{30}H_{38}O_{15}$	0.01	$[M-H]^-$	637.2138	491.1571,475.1824, 461.1664,193.0506, 179.0351,175.0359, 161.0246	Phenylpropanoid	✓	✓	✓	✓	✓	✓
**107**	34.546	Ningposide I	$C_{30}H_{40}O_{16}$	0.65	$[M+Na]^+$	679.2213	661.2101,382.1652, 163.0387,149.0599, 130.9662	Iridoid	✓	✓	✓	✓	✓	✓
**108**	35.268	Verbascoside A	$C_{31}H_{40}O_{16}$	-0.05	$[M+HCOO]^-$	713.2298	361.1502,160.8440	Iridoid	✓	✓	✓	✓	✓	✓

**109**	35.926	Scorodioside	$C_{32}H_{40}O_{16}$	-0.22	$[M+Na]^+$	703.2207	541.1692,523.1578, 375.1049	Iridoid	✓	✓	✓	✓	✓	✓
**110**	36.489	Martynoside	$C_{31}H_{40}O_{15}$	0.25	$[M+HCOO]^-$	697.2351	551.1757,457.1844	Phenylpropanoid	✓	✓	✓	✓	✓	✓
**111**	36.74	1-Octen-3-yl-primeveroside	$C_{19}H_{34}O_{10}$	-3.31	$[M+HCOO]^-$	467.2143	289.1658,161.0454, 131.0338	Others	✓	✓	✓	✓	✓	✓
**112**	37.031	Acetylangoroside C	$C_{38}H_{50}O_{20}$	0.63	$[M+NH4]^+$	844.3239	339.1081,321.1168, 195.0654,177.0547	Phenylpropanoid	✓	✓	✓	✓	✓	✓
**113**	37.497	Cistanoside D	$C_{31}H_{40}O_{15}$	-0.07	$[M-H]^-$	651.2294	160.8429,145.0499	Phenylpropanoid	✓	✓	✓	✓	✓	✓
**114**	38.428	6′-O-Cinnamoylharpagide	$C_{24}H_{30}O_{11}$	0.13	$[M+Na]^+$	517.1681	499.1576,333.0944, 207.0621,131.0488	Iridoid	✓	✓	✓	✓	✓	✓
**115**	38.522	Scrophuloside A3	$C_{32}H_{40}O_{17}$	-0.97	$[M-H]^-$	695.2186	533.1667,515.1569, 181.0505,163.0398, 145.0295,	Iridoid	✓	✓		✓	✓	✓
**116**	38.77	*cis*-Martynoside	$C_{31}H_{40}O_{15}$	0.25	$[M+HCOO]^-$	697.2351	551.1784	Phenylpropanoid	✓	✓	✓	✓	✓	✓
**117**	38.893	6-O-*α*-*D*-Galactopyranosyl harpagoside	$C_{30}H_{40}O_{16}$	0.8	$[M+Na]^+$	679.2214	365.1234,149.0599, 130.9662,	Iridoid	✓	✓	✓	✓	✓	✓

**118****	39.076	Harpagoside	$C_{24}H_{30}O_{11}$	-1.03	$[M+Na]^+$	517.1675	369.1155,351.1051, 203.0526,189.0523, 149.0600	Iridoid	✓	✓	✓	✓	✓	✓
**119**	39.361	8-O-Methoxycinnamoyl harpagide	$C_{25}H_{32}O_{12}$	2.2	$[M+Na]^+$	547.1798	369.1155,203.0524, 149.0593	Iridoid	✓	✓	✓	✓	✓	✓
**120**	39.661	Eurostoside	$C_{24}H_{28}O_{11}$	1.39	$[M+Na]^+$	515.1531	169.0863,167.0701	Iridoid	✓	✓	✓	✓	✓	✓
**121**	39.755	*Z*-Harpagoside	$C_{24}H_{30}O_{11}$	0.71	$[M+HCOO]^-$	539.1774	201.1128,160.8417, 147.0452	Iridoid	✓	✓	✓	✓	✓	✓
**122**	40.006	Scrophuloside A5	$C_{33}H_{42}O_{17}$	0	$[M+HCOO]^-$	755.2404	547.1779,343.1053, 325.0921,181.0495, 177.0560,145.0282	Iridoid	✓	✓	✓	✓	✓	✓
**123**	40.135	Buddlejoside A2	$C_{33}H_{42}O_{16}$	-0.57	$[M+Na]^+$	717.2361	675.2143,537.1727, 389.1205,203.0523, 161.0586	Iridoid	✓	✓	✓	✓	✓	✓
**124****	40.139	Cinnamic acid	$C_9H_8O_2$	0.32	$[M-H]^-$	147.0452	103.0552	Organic acid	✓	✓	✓	✓	✓	✓
**125**	40.812	Diacetylmartynoside	$C_{35}H_{44}O_{17}$	1.53	$[M-H]^-$	735.2517	517.3176,329.2234, 311.1683	Phenylpropanoid	✓	✓	✓	✓	✓	✓
**126**	40.955	Massbank:PR309108 FA 18:1+3O	$C_{18}H_{34}O_5$	-0.75	$[M-H]^-$	329.2331	182.9885,138.9696	Others	✓	✓	✓	✓	✓	✓

**127**	41.006	Lauryldiethanolamine	$C_{16}H_{35}NO_2$	0.53	$[M+H]^+$	274.2742	230.2119,102.1275	Others	✓	✓	✓	✓	✓	✓
**128**	41.436	Scropolioside B	$C_{41}H_{46}O_{17}$	0.35	$[M+HCOO]^-$	855.272	679.2950,531.3013, 517.3169,407.1870, 325.1861,227.0498, 160.8415	Iridoid	✓	✓	✓	✓	✓	✓
**129**	41.806	Lauramidopropyl betaine	$C_{19}H_{38}N_2O_3$	0.53	$[M+H]^+$	343.2957	281.2478,108.9618	Others	✓	✓	✓	✓	✓	✓
**130**	42.229	Scrophuloside A9	$C_{41}H_{48}O_{18}$	0.04	$[M+HCOO]^-$	873.2823	693.2970,487.3437, 327.2182,160.8435	Iridoid	✓	✓	✓	✓	✓	✓
**131**	43.657	Scrophuloside A4	$C_{43}H_{50}O_{19}$	1.17	$[M+HCOO]^-$	915.2939	647.2109,593.2033, 181.0507,177.0562, 163.0406,161.0453	Iridoid	✓	✓	✓	✓	✓	✓
**132**	43.945	Scrophuloside B4	$C_{42}H_{48}O_{18}$	0.04	$[M+HCOO]^-$	885.2823	677.2240,605.1986, 181.0507,177.0556, 147.0452,145.0290	Iridoid	✓	✓	✓	✓	✓	✓
**133**	44.126	Methoxylscrophuloside B4	$C_{43}H_{50}O_{19}$	0.05	$[M-H]^-$	869.2874	549.1769,177.0557, 161.0460,143.0336	Iridoid	✓	✓	✓	✓	✓	✓
**134**	44.536	Massbank:PR310841 LPC 18:3	$C_{26}H_{48}NO_7P$	0.16	$[M+H]^+$	518.3242	104.1069	Others	✓	✓		✓	✓	✓
**135**	44.985	Acetyl tributyl citrate	$C_{20}H_{34}O_8$	1.13	$[M+H]^+$	403.2331	185.0796	Organic acid	✓	✓	✓	✓	✓	✓

**136**	45.671	PE (18:2/0:0)	$C_{23}H_{44}NO_7P$	-2.44	$[M-H]^-$	476.2771	196.0253,140.0259	Others	✓	✓		✓	✓	✓
**137**	46.027	Massbank:PR309158 LPC 18:2	$C_{26}H_{50}NO_7P$	-0.52	$[M+HCOO]^-$	564.3304	504.3078,281.2470	Others	✓	✓	✓	✓	✓	✓
**138**	47.215	1-Palmitoyl-2-hydroxy-sn-glycero-3-phosphoethanolamine	$C_{21}H_{44}NO_7P$	0.08	$[M-H]^-$	452.2783	255.2325,112.9860	Others	✓	✓	✓	✓	✓	✓
**139**	47.322	Lyso-PC (16:0)	$C_{24}H_{50}NO_7P$	0.87	$[M+H]^+$	496.3402	104.1069	Others	✓	✓	✓	✓	✓	✓
**140**	47.792	1-(9*Z*-Octadecenoyl)-sn-glycero-3-phosphoethanolamine	$C_{23}H_{46}NO_7P$	-0.45	$[M-H]^-$	478.2937	281.2504,196.0249	Others	✓	✓	✓	✓	✓	✓
**141**	47.905	1-(9*Z*-Octadecenoyl)-sn-glycero-3-phosphocholine	$C_{26}H_{52}NO_7P$	0.44	$[M+Na]^+$	544.3376	485.1121,104.1071	Others				✓	✓	✓
**142**	48.008	Massbank:PR309160 LPC 18:1	$C_{26}H_{52}NO_7P$	0.28	$[M+HCOO]^-$	566.3465	281.2496	Others	✓	✓	✓	✓	✓	✓
**143**	48.067	Scrodentoid B	$C_{20}H_{26}O_3$	2	$[M+H]^+$	315.1961	297.0822,279.1010	Others	✓	✓	✓	✓	✓	✓
**144**	48.632	Sugiol	$C_{20}H_{28}O_2$	-0.35	$[M+H]^+$	301.2161	283.2035,259.1692	Others	✓	✓	✓	✓	✓	✓

**145**	49.754	Oleic acid	$C_{18}H_{34}O_2$	1.05	$[M-H]^-$	281.2489	218.943	Others			✓	✓	✓	✓
**146**	49.761	1-Hexadecanoyl-sn-glycero-3-phospho-(1'-myo-inositol)	$C_{25}H_{49}O_{12}P$	1.25	$[M-H]^-$	571.2896	255.2327	Others		✓	✓	✓	✓	✓
**147**	51.165	Dibutyl Phthalate	$C_{16}H_{22}O_4$	2.2	$[M+H]^+$	279.1597	149.0235,122.9637	Others	✓	✓	✓	✓	✓	✓
**148**	51.252	Phthalic Anhydride	$C_8H_4O_3$	1.2	$[M+H]^+$	149.0235	122.964	Others	✓	✓	✓	✓	✓	✓
**149**	51.475	Ursolic Acid	$C_{30}H_{48}O_3$	0.29	$[M-H]^-$	455.3532	375.2953	Others		✓	✓	✓	✓	✓

*Compounds which are identified by inferring from GNPS and MS^2 fragment ions; **Compounds which are identified by comparing with standards; Compounds which are identified by comparing with the self-built database and GNPS.
